# Supplementary material for: Association between physical activity levels in mid-life with physical activity in old age: a 20-year tracking study in a prospective cohort
Source: BMJ Open. 2017 Sep 21;7(8):e017378. doi: 10.1136/bmjopen-2017-017378 (PMC5724234; doi:10.1136/bmjopen-2017-017378)
Supplement: Supplementary file 1 [file bmjopen-2017-017378supp001.pdf]

Supplementary table 1. Stability of physical activity variables over time by changes in working status (n=3288)

	Wave 1 to 2	Wave 1 to 3	Wave 1 to 4
	Kappa	Kappa	Kappa
**Total Physical Activity**			
No change in working status	0.28	0.18	0.24
Retired between follow ups	0.25	0.25	0.25
**Sport participation**			
No change in working status	0.36	0.32	0.33
Retired between follow ups	0.40	0.35	0.35
**Recreational activity**			
No change in working status	0.26	0.18	0.27
Retired between follow ups	0.22	0.19	0.14
**Walking**			
No change in working status	0.18	0.16	0.13
Retired between follow ups	0.12	0.09	0.11

Note. Kappa statistics are presented for participants with a valid physical activity score at all four time points and valid data on working status n=3288. 46.4% (n=1526) of men retired between wave 1 and 2; 71.5% (n=2352) of men retired between wave 1 and 3; and 79.4% (n=2611) were retired between wave 1 and 4.
