# Supplementary material for: Association between physical activity levels in mid-life with physical activity in old age: a 20-year tracking study in a prospective cohort
Source: BMJ Open. 2017 Sep 21;7(8):e017378. doi: 10.1136/bmjopen-2017-017378 (PMC5724234; doi:10.1136/bmjopen-2017-017378)
Supplement: Author's manuscript [file bmjopen-2017-017378.draft_revisions.pdf]

BMJ Open is committed to open peer review. As part of this commitment we make the peer review history of every article we publish publicly available.

When an article is published we post the peer reviewers' comments and the authors' responses online. We also post the versions of the paper that were used during peer review. These are the versions that the peer review comments apply to.

The versions of the paper that follow are the versions that were submitted during the peer review process. They are not the versions of record or the final published versions. They should not be cited or distributed as the published version of this manuscript.

BMJ Open is an open access journal and the full, final, typeset and author-corrected version of record of the manuscript is available on our site with no access controls, subscription charges or pay-per-view fees (http://bmjopen.bmj.com).

If you have any questions on BMJ Open's open peer review process please email info.bmjopen@bmj.com

Association between physical activity levels in midlife with physical activity in old age: A 20-year tracking study

Journal:	*BMJ Open*
Manuscript ID	bmjopen-2017-017378
Article Type:	Research
Date Submitted by the Author:	21-Apr-2017

Complete List of Authors:	Aggio, Daniel; University College London, Department of Primary Care and Population Health<br>Papacosta, Olia; Department of Primary Care and Population Health London, UK<br>Lennon, Lucy; UCL, Department of Primary Care and Population Health<br>Whincup, Peter; St Georges, University of London, Population Health Research Institute<br>Wannamethee, Goya; University College London, Department of Primary Care and Population Health<br>Jefferis, Barbara ; University College London, Department of Primary Care and Population Health

**Primary Subject Heading**:	Epidemiology
Secondary Subject Heading:	Public health, Sports and exercise medicine
Keywords:	EPIDEMIOLOGY, PREVENTIVE MEDICINE, PUBLIC HEALTH

SCHOLARONE™ Manuscripts

Association between physical activity levels in midlife with physical activity in old age: A 20-year tracking study

Aggio, Daniel[1,2]; Papacosta, Olia[1]; Lennon, Lucy T.[1]; Whincup, Peter H.[3]; Wannamethee S. Goya[1;] Jefferis, Barbara J.[1,2]

[1.] UCL Department of Primary Care & Population Health, UCL Medical School, London, United Kingdom

[2.] UCL PA Research Group, London, United Kingdom

[3.] Population Health Research Institute, St George's University of London, Cranmer Terrace, London, United Kingdom

Corresponding author: Daniel Aggio, UCL Department of Primary Care & Population Health, UCL Medical School, Rowland Hill Street, London NW3 2PF, UK.

Email d.aggio@ucl.ac.uk

Tel +44 207 830 2335

Fax: +44 20 7472 6871

Word count: 3920, 4 tables and 1 supplementary table

Keywords: aging, sport, physical activity

For peer review only

ABSTRACT

Objectives:

This study aims to examine the tracking and predictability of physical activity in old age from overall physical activity and participation in sport, recreational activity and walking in midlife.

Setting:

British Regional Heart Study participants recruited from Primary Care Centres in the United Kingdom in 1978-80.

Participants and outcome measures:

Men (n=3413) self-reported their physical activity at baseline, 12-, 16- and 20-year follow ups and were categorised as inactive or active and having high or low participation in sport, walking and recreational activities. Tracking was assessed using kappa statistics and random effects models. Logistic regression estimated the odds of being active at 20-year follow up according to physical activity participation in midlife.

Results:

Among 3413 men (mean age at baseline 48.6 ± 5.4 years) with complete data, tracking of overall physical activity was moderate (kappa: 0.23-0.26). Tracking was higher for sports participation (kappa: 0.35-0.38) compared to recreational activity (kappa: 0.16-0.24) and walking (kappa: 0.11-0.15). Intraclass correlation coefficients (ICCs) demonstrated similar levels of stability and only marginally weakened after controlling for covariates. Compared to inactive men, being active at baseline was associated with greater odds of being active at 20-year follow up (odds ratio (OR) 2.7, 95% confidence interval (CI), 2.4, 3.2) after adjusting

For peer review only

for sociodemographic, health and lifestyle variables. Playing sport in midlife was more strongly associated with being active at 20-year follow up than other domains, particularly when sport participation begun earlier in life.

Conclusion:

Being physically active in midlife increases the odds of being active in old age. Promoting physical activity in later life might be best achieved by promoting sport participation earlier in the lifecourse.

Strengths and limitations of this study

- This study investigates the tracking of overall and specific domains of physical activity during the transition to old age over 20 years, an understudied period of the lifecourse.
- Very few studies have investigated the tracking of specific domains of physical activity during this period
- The main limitation of this study is the self-reported assessment of physical activity, which may have been prone to recall bias
- Our results may also not be generalizable to women and non-white ethnic groups

Introduction

For peer review only

Prospective epidemiological studies have shown that physical activity (PA) in midlife and old age is associated with numerous health benefits, including reductions in cardiovascular disease (CVD) events and mortality [1, 2, 3]. Taking up PA in later life may reduce the risk of adverse health outcomes, but maintaining a physically active lifestyle throughout the life course may provide optimal health benefits [4, 5, 6]. The transition from midlife to old age typically coincides with major life events (e.g. retirement) and therefore may be an important window when both the volume and type of PA are likely to change. Knowledge on the stability, or tracking, of PA during this transition is very limited. The tracking of a behaviour over time can be determined by 1) its stability of over time, typically estimated using correlations between repeated measures or 2) the predictability of later measures from previous ones [7]. Past exercise behaviour is a consistent predictor of current PA levels [8]; however, few studies have examined the predictability of PA in old age from PA measures in midlife. Understanding tracking of PA during this transition may help inform interventions aiming to promote or maintain activity levels from midlife to old age.

There is a large body of research on the tracking of PA from childhood, but few studies have extended over prolonged periods in adults[9]. Current evidence suggests low to moderate tracking of PA throughout the lifecourse [9, 10, 11]. Studies tracking physical activity in youth have shown that sport participation in early life tracks more strongly [9] and is a stronger predictor of activity levels in adulthood (age 42 years) compared to other domains of activity such as outdoor play [12]. However, tracking studies in adults have rarely distinguished between the type of physical activity. Thus, it remains unknown what types of activity in midlife are more likely to predict PA in old age. The limited evidence in older adults has suggested some domains of PA are more liable to change (e.g. indoor activities) than others (e.g. outdoor and leisure activities) [13] and thus may be easier to modify. Further studies have

For peer review only

investigated the predictability of activity levels in early old age according to PA in early adulthood. For example, one study showed that being moderately active in young adulthood (mean age 35 years) increased the odds of being active 28 years later by more than three times [14]. Another study showed that sport participation in healthy young men (aged 25 years) strongly predicted PA 50 years later [15]. However, this study was retrospective in nature and may not be generalizable to less healthy populations.

Overall, very few tracking studies have extended into old age. Furthermore, the predictability of PA in later life from participation in specific types of activity in midlife remains unknown. Thus we aimed to estimate the tracking of overall and specific domains of PA from midlife to old age and the predictability of PA levels in old age from 1) overall PA and 2) PA domains in midlife.

Methods

Participants

Data were drawn from the British Regional Heart Study an ongoing prospective cohort study involving 7735 men (response rate = 78%) from 24 towns in Great Britain [16]. Men were recruited from primary care practices and were first examined in 1978-80 aged 40-59 years and were followed up after 12, 16 and 20 years. Response rates for surviving cohort members were 91% (n=5925), 88% (n=5263) and 77% (n=4252) at 12-, 16- and 20-year follow ups, respectively. Men completed a lifestyle and medical history questionnaire at the time of the examination (baseline and 20-year follow up) or by post (12- and 16-year follow ups). Participants provided informed written consent to the investigation. Ethical approval was obtained from the National Research Ethics Service (NRES) Committee London.

For peer review only

Measures

Self-reported PA

At all waves, participants reported their usual PA levels. Questions referred to journeys made by foot and the time spent on these journeys, how long spent on recreational activities (such as gardening, chores, do-it-yourself (DIY) and how frequently they participate in sport/exercise. Responses to each domain of PA were scored based on the intensity and frequency of the activity [17, 18]. For example, making no journeys by foot was scored as 0 and >90 minutes/weekday was scored as 5. Scores were also heavily weighted for vigorous activities. For example, playing sport 4-7 times a month was given a score of 8. Scores for each domain were summed together to give a total PA score. The original scoring system has been reported in detail elsewhere [19]. The total PA score was then used to classify activity levels as inactive, occasional, light, moderate, moderately vigorous or vigorous. These PA scores have previously been validated against heart rate, forced expiratory volume in 1 second [19] and objectively measured PA[20]. For the purposes of this study the categories were grouped into active or inactive (inactive and occasional groups were classified as inactive). Responses to individual questions were also used to classify participation in specific domains of activity. Men were classified as having high or low sport/exercise participation (no sport/exercise participation was classified as low), high or low walking (low walking was classified as ≤20 mins/day) and high or low recreational activity (low recreational activity was defined as being similar or less active than someone who spends two hours on most days on recreational activities). Men who reported participating in sport also retrospectively disclosed how many years they had been involved in that activity, from which men were classified as participating in sport for ≤ 4 years, 5-11 years, 12-24 years and ≥ 25 years.

For peer review only - http://bmjopen.bmj.com/site/about/guidelines.xhtml

Covariates

Participants self-reported their age at baseline; social class, which was derived from their longest held occupation [21] and categorised as manual or non-manual; and cigarette smoking habits, classified as current or ex-smokers and never smokers. Nurses measured participant's height and weight, which was used to derive body mass index. Men were then categorised as overweight or obese (Body Mass Index [BMI]: ≥25.0 Kg/m2) or healthy weight (BMI:<25.0 Kg/m2).

Statistical analysis

Descriptive statistics were used to report sample characteristics at baseline and the proportion of men active/high participation at each wave. McNemar's chi squared test was used to determine whether the proportion reporting being physically active changed between time points. Cohen's kappa was used to assess the observed agreement compared with the expected agreement. We followed suggestions by Munoz and Bangdiwala for interpretation of K coefficients : <0.00 indicates poor agreement, 0.00-0.20 fair agreement, 0.21-0.45 moderate agreement, 0.46-0.75 substantial agreement and 0.76-1.0 indicates near perfect agreement [22]. Random effects models were also used to calculate intraclass correlation coefficients (ICCs), providing an indicator of tracking using data from all assessments whilst also controlling for covariates. In a supplementary analysis, we stratified our sample according to changes in employment status as we hypothesised that the timing of retirement may affect the stability of PA. We categorised men as no change in employment status (representing continuous employment/seeking employment and continuously retired) or

For peer review only

retiring (i.e. retired between baseline and the respective follow up) and presented kappa statistics separately. Finally, we used logistic regression to estimate the odds ratio for being active compared to being inactive at 20-year follow up according to 1) overall activity levels at baseline, 2) engagement in specific domains of PA at baseline and 3) duration of sports participation. Tests for linear trend were also conducted by entering sports duration as a continuous variable into regression models. Initial models were adjusted for age, entered as a continuous variable (model 1) and then for BMI, social class and smoking status (categorical) (model 2). In analyses using just baseline activity levels as predictors of activity (i.e. not sports duration) at 20-year follow up, we also introduced a third model including all PA variables to identify the strongest predictor of activity 20 years later whilst also accounting for participation in other types of PA.

For peer review only

Results

7735 men responded to the baseline survey. Men who died during follow up (26.6%, n=2060), those with missing PA data (29.1%, n=2251) at one or more examination between baseline and 20-year follow up and those with missing covariate data (0.1%, n=11) were excluded from analyses, leaving 3413 for analyses. Compared to men in the analytic sample, men excluded from the analyses were significantly older (baseline age, 48.6 vs. 51.5 years, $p<0.001$), had a higher BMI (baseline BMI, 25.3 vs. 25.7, $p<0.001$) and were more likely to be inactive at baseline (proportion inactive at baseline, 55.5% vs. 66.1%, $p<0.001$). A larger sample was included in the random effects models, as men were only excluded if they did not provide PA measures on at least two assessments and have valid covariate data.

Table 1 displays sample characteristics and the proportion of men who were physically active and who participated in PA domains at each time point. Between baseline and 12-year follow

up the number of men classified as active increased from 66.1% to 71.0% (p<0.001) and then dropped significantly to 63.7% and 66.9% (p<0.001) at 16- and 20-year follow ups, respectively. The proportion of men reporting high levels of walking increased from 37.9% at baseline to 68.2% at 20-year follow up (p<0.001). There were also steep declines over the 20-year follow up in recreational activity, with 56.0% of men reporting high levels of recreational activity at baseline and 40.2% at 20-year follow up.

For peer review only

Table 1. Sample characteristics and physical activity levels at baseline, 12-, 16- and 20-year follow up, n=3413

	Baseline	12 year	16 year	20 year
Age (years, mean ± SD)	48.6 ± 5.4	62.2 ± 5.4	66.2 ± 5.4	68.5 ± 5.4
Overweight/Obese (%, n)	52.2 (1783)			
Current smoker (%, n)	30.6 (1043)			
Manual Occupation (%, n)	50.2 (1713)			
Physically active[a] (%, n)	66.1 (2257)	71.0 (2422)	63.7 (2173)	66.9 (2284)
High sport participation[b] (%, n)	47.7 (1627)	45.3 (1532)	44.6 (1493)	49.2 (1663)
High recreational activity[c] (%, n)	56.0 (1912)	58.4 (1994)	41.2 (1407)	40.2 (1372)
High walking[d] (%, n)	37.9 (1292)	63.5 (2157)	63.5 (2165)	68.2 (2328)

Note. Data presented are for participants with a valid physical activity score at all four time points (n=3413). Data on walking was missing for an additional 15 participants at 12 year follow up, 3 participants at 16-year follow up and 1 participant at 20-year follow up. Data on sport participation was missing for 33 participants at 12 year follow up, 68 participants at 16 year follow up and 34 participants at 20 year follow up.

[a] Physically active was classified as reporting at least light activity.

[b] High sport was classified as reporting at least occasional participation (less than once a month)

[c] high walking was classified as >20 mins/day

[d] high recreational activity was classified as >2hours/day on recreational activities

Table 2 presents kappa statistics and ICC for PA variables. Kappa statistics for overall PA ranged from 0.23-0.26 between baseline and subsequent time points, but were highest for sports participation (0.35-0.38) and lowest for walking (0.10-0.16). Kappa statistics were generally higher for shorter follow up periods. In random effects models, ICCs were consistent with the Kappa statistics and were only marginally weakened after controlling for covariates. In a supplementary analysis, we present Kappa statistics according to employment status. Overall stability of total PA was similar between men who reported no change in working status and men who retired between baseline and subsequent follow ups (see Supplementary Table 1). However, a higher proportion of men who were retiring increased their total activity between baseline and subsequent follow ups compared to men who reported no change in their working status (e.g. 21.3% vs. 15.7% of men increased their total activity levels between Wave 1 and Wave 2 in the retiring group and the no change group, respectively [data not shown]). Similarly, the overall stability of sport participation was comparable between men who reported no change in working status and retiring men, but the retiring group contained a higher proportion of men who increased their sport participation (e.g. 15.8% vs. 12.6% of men increased their sports participation between Wave 1 and Wave 2 in the retiring group and the no change group, respectively [data not shown]). Stability of recreational activity was markedly lower in men retiring between baseline and wave 4 compared to men who reported no change in their working status during the same period. This was largely explained by a higher proportion of retiring men reporting a decrease in recreational activity compared to men reporting no change in work status (e.g. 30.4% vs. 24.7% of men reported a decrease in recreational activity between Wave 1 and Wave 4 in the retiring group and the no change group, respectively). There were also some clear differences in the stability of walking activity between men who reported no change in working status and retiring men. This was largely explained by a higher proportion of retiring men reporting

For peer review only

an increase in walking activity compared to men with no change in working status (e.g. 39.1% vs. 32.3% of men reported an increase in walking activity between Wave 1 and Wave 2 in the retiring group and the no change group, respectively).

For peer review only

35 Table 2. Stability of physical activity variables over time, n=3413

	Wave 1 to 2	Wave 1 to 3	Wave 1 to 4	Random Effects Models	
				Univariate	Multivariate[a]
	Kappa	Kappa	Kappa	ICC (95% CI)	ICC (95% CI)
Physically activity	0.26	0.23	0.24	0.46 (0.43, 0.48)	0.44 (0.41, 0.46)
Sport participation	0.38	0.35	0.35	0.65 (0.63, 0.67)	0.61 (0.59, 0.63)
Recreational activity	0.24	0.19	0.16	0.38 (0.36, 0.40)	0.36 (0.34, 0.39)
Walking	0.15	0.11	0.11	0.31 (0.29, 0.33)	0.31 (0.29, 0.33)

36 *Note.* Kappa statistics are presented for participants with a valid physical activity score at all four time points (n=3413). Data on walking was
37 missing for and additional 15 participants at 12 year follow up, 3 participants at 16-year follow up and 1 participant at 20-year follow up. Data
38 on sport participation was missing for 33 participants at 12 year follow up, 68 participants at 16 year follow up and 34 participants at 20 year
39 follow up. Random Effects Models included men with at least two assessments for each domain accompanied by valid covariate data (Physical
40 activity: n= 5962; Sport participation: n= 6122; Recreational activity: n=6093; Walking: n=6040). ICC = Intraclass correlation coefficients from
41 random effects models

42 [a] adjusted for age, BMI, social class and smoking status at baseline

For peer review only

Compared to inactive men, being physically active at baseline was associated with greater odds (OR 2.7, 95% CI, 2.4, 3.2) of being physically active at 20-year follow up after adjusting for age, social class, BMI and smoking status at baseline (Table 3). Odds ratios for being active at 20-year follow up were similarly raised for men who played sport at baseline after adjustments (OR 2.7 95% CI, 2.3, 3.1). High participation in walking and recreational activity at baseline were also associated with greater odds of being active at 20-year follow up (OR: 1.3-1.6). In the final model including all PA domains, sport participation at baseline remained the strongest predictor of being active at 20-year follow up.

Table 3. Odds of being active at 20 year follow up according to activity levels at baseline, n=3413

		Model 1	Model 2	Model 3
	N	OR (95% CI)	OR (95% CI)	OR (95% CI)
Physical activity				
Inactive	1,156	1.0	1.0	–
Active	2,257	2.9 (2.5, 3.3)	2.7 (2.4, 3.2)	–
Sport				
Low	1,786	1.0	1.0	1.0
High	1,627	2.9 (2.5, 3.4)	2.8 (2.4, 3.3)	2.7 (2.3, 3.1)
Recreational activity				
Low	1,501	1.0	1.0	1.0
High	1,912	1.9 (1.6, 2.2)	1.8 (1.6, 2.1)	1.6 (1.4, 1.9)
Walking				
Low	2,121	1.0	1.0	1.0
High	1,292	1.4 (1.2, 1.6)	1.4 (1.2, 1.6)	1.3 (1.2, 1.6)

Model 1 adjusted for baseline age. Model 2 additionally adjusted for social class, BMI and smoking status at baseline. Model 3 mutually adjusted for all domains of activity respectively.

For peer review only

Table 4. Odds of being active at 20 year follow up according to duration of sport participation at baseline (n=2969)

		Model 1	Model 2
	N	OR (95% CI)	OR (95% CI)
Sports participation duration			
Not participating at baseline	1786	1.0	1.0
≤ 4 years	262	2.4 (1.8, 3.2)	2.3 (1.7, 3.2)
5-11 years	331	3.0 (2.2, 4.0)	2.9 (2.2, 3.9)
12-24 years	290	4.4 (3.1, 6.2)	4.3 (3.1, 6.0)
≥ 25 years	300	5.0 (3.6, 7.1)	4.8 (3.4, 6.8)
p value for trend[a]		<0.001	<0.001

Model 1 adjusted for baseline age. Model 2 additionally adjusted for social class, BMI and smoking status at baseline.

[a] Tests for linear trend were conducted by entering sports duration as a continuous variable into regression models

Table 4 shows the odds of being active at 20-year follow up according to duration of sports participation from baseline. This sample size was lower than the main analytic sample as 27.3% (n=444) of men who played sport did not report duration of participation. Longer duration of sports participation was associated with increased odds of being active at 20-year follow up. Compared to those who were not participating in sport at baseline, taking part in sport for 25 years or more was most strongly associated with being active at 20-year follow up (OR 4.8, 95% CI, 3.4, 6.8). However, even taking up sport recently (≤ 4 years) was associated with increased odds of being active at 20-year follow up (OR 2.3, 95% CI, 1.7, 3.2).

Discussion

This study investigated the tracking of overall and specific domains of PA from midlife to old age and the predictability of PA in old age from overall PA and participation in sport,

For peer review only

recreational activity and walking in midlife. Agreement between overall PA levels at baseline and subsequent measures at 12-, 16- and 20-year follow ups suggested moderate levels of tracking, with stronger tracking evident for sport participation compared to other domains. Importantly, however, prevalence of walking >20 mins/day increased from around a third to approximately two thirds by 20-year follow up. Comparisons with previous studies are challenging given the various measures, cut off points and time frames studied, but our findings appear to be in agreement with previous studies in similar age groups [9, 23, 24], but over a longer time-span. As expected, tracking coefficients tended to be higher for the shorter follow up periods.

This is the first study that we are aware of to examine tracking of specific domains of activity from midlife to old age and the predictability of PA in old age from PA domains in midlife. Sports participation was the most stable domain with moderate agreement between baseline and subsequent time points. Tracking was fair for walking owing to a high proportion increasing their walking activity (e.g. 39.2% of retired men increased from low to high walking between baseline and 12-year follow up). Tracking of recreational activity was fair to moderate but a large proportion decreased their recreational activity by 20-year follow up (e.g. 30.5% of retired men decreased from high to low recreational activity between baseline and 12-year follow up). Tracking indicators from random effects models provided comparable estimates of tracking, whilst using all available measurements and controlling for factors that may influence tracking. The differential changes over time between domains of activity may reflect the changing opportunities and functional abilities associated with ageing. Our supplementary analyses suggest that retirement may be a period when PA behaviours are more sensitive to change. Increased free time during retirement may possibly explain the observed steep increase in walking and lower levels of stability during this transition, whereas declines in physical function and onset of chronic disease may explain

For peer review only

reductions in the more strenuous activities related to recreational activity, such as DIY and gardening.

PA during midlife was associated with increased odds of being active in old age. Comparable results were found in a study by Morseth et al., [14], who found that Norwegian men and women who were active in midlife (aged 20-54 at baseline) were 5 to 13 times more likely to remain non-sedentary 28 years later. Although similar, our findings come from a sample of older British men all of whom would have transitioned to old age over the 20-year period and would take part in very different activities to their Norwegian counterparts. Sport participation in midlife predicted PA in old age more strongly than walking and recreational activity. This is consistent with previous tracking studies from childhood to adulthood that have also shown that sporting activity tracks more strongly [9] and is a stronger predictor of PA later in life than other domains of activity [12, 25]; however, this is the first study that we are aware of to demonstrate similar findings during the transition to old age. There may be a number of reasons why participation in sport in midlife more strongly predicts activity in older age than other types of activity. One possibility is that people's enjoyment of sport may be more likely to persist into old age than preferences for other types of activity. Sport participation in midlife may help maintain physical function and PA self-efficacy in later life, increasing psychological and physical readiness for PA in old age. Stronger levels of tracking may also suggest that participation in sport is less modifiable than other domains. By contrast, lower levels of tracking for walking, predominantly caused by large increases in uptake, suggest that walking may be easier to adopt in older adults, particularly those with functional limitations. Thus, improving our understanding on how to promote this domain of PA is important for future research. We also found that earlier engagement in sport more strongly predicted PA in old age. Early engagement in sport may be crucial for establishing a lifelong habit for sport participation. Further, we know that childhood is a critical time for

For peer review only

developing motor skills. Thus earlier engagement may result in improved capability to maintain PA and sport in older age. Although earlier sport participation appears favourable, even taking up sport in midlife more than doubled the odds of being active in old age compared to adults not taking part in sport in midlife. Encouraging sport participation early in the lifecourse may be most effective for promoting life-long PA but even interventions promoting uptake in middle-aged adults may be successful.

The main strengths of this study are its large sample size and long follow up, encompassing the transition from midlife to old age, an understudied period in PA tracking studies. Random effects models also allowed estimation of tracking using all available data, whilst also accounting for factors that may influence tracking strength. Our study is limited by the use of self-reported assessment of PA, which may have been prone to bias. However, the questionnaire was validated at baseline against heart rate and forced expiratory volume in 1 second [19] and more recently against objectively measured PA [20]. Use of the same questionnaire at successive waves should result in comparable results between waves. Self-reports also allowed us to examine how specific types of PA track, which may provide useful insight for intervention strategy. Another limitation is that our findings may not be generalizable to women and non-white ethnic groups, who are not represented in this study sample.

Conclusion

In conclusion, PA tracks moderately from midlife to old age. Being active in midlife was associated with increased odds of being active in old age. Playing sport in midlife was more strongly associated with PA in old age than other domains of PA. Encouraging early and sustained sports participation into midlife may be effective for promoting PA in old age, but

For peer review only

increased opportunities to take up other forms of activity, such as walking, may also be crucial as people transition into old age.

Footnotes

Contributors: SGW and PW designed and conceived the study. DA analysed and interpreted the data and drafted the initial manuscript. LL collected the data. OP generated the database. BJJ SGW interpreted the data and revised the manuscript. OP SGW PW LL BJJ DA approved the final manuscript.

Funding: DA is funded by a British Heart Foundation PhD studentship. This research was also supported by an NIHR Post-Doctoral Fellowship awarded to BJJ (2010–03–023) and by a British Heart Foundation project grant (PG/13/86/30546) to BJJ. The British Regional Heart study is supported by a British Heart Foundation grant (RG/13/16/30528).

Competing interests: None declared.

Ethical approval: Participants provided informed written consent to the investigation. Ethical approval was obtained from the National Research Ethics Service (NRES) Committee London.

Provenance and peer review: Not commissioned; externally peer reviewed.

Data sharing statement: Data are not publically available, but applications for data sharing can be made. For enquiries please contact Lucy Lennon (l.lennon@ucl.ac.uk).

For peer review only

References

1. Wannamethee SG, Shaper AG, Walker M, et al. Lifestyle and 15-year survival free of heart attack, stroke, and diabetes in middle-aged British men. Arch Intern Med 1998;158:2433-40.

2. Nocon M, Hiemann T, Muller-Riemenschneider F, et al. Association of physical activity with all-cause and cardiovascular mortality: a systematic review and meta-analysis. Eur J Cardiovasc Prev Rehabil 2008;15:239-46.

3. Li J, Siegrist J. Physical activity and risk of cardiovascular disease--a meta-analysis of prospective cohort studies. Int J Environ Res Public Health 2012;9:391-407.

4. Wannamethee SG, Shaper AG, Walker M. Changes in physical activity, mortality, and incidence of coronary heart disease in older men. Lancet 1998;351:1603-8.

5. Stessman J, Hammerman-Rozenberg R, Cohen A, et al. Physical Activity, Function, and Longevity Among the Very Old. Archives of Internal Medicine 2009;169:1476-83.

6. Byberg L, Melhus H, Gedeborg R, et al. Total mortality after changes in leisure time physical activity in 50 year old men: 35 year follow-up of population based cohort (Reprinted from BMJ, vol 338, b688, 2009). Brit J Sport Med 2009;43:482-U25.

7. Twisk JWR. Applied Longitudinal Data Analysis for Epidemiology A Practical Guide: Cambridge University Press; 2003.

8. Trost SG, Owen N, Bauman AE, et al. Correlates of adults' participation in physical activity: review and update. Med Sci Sport Exer 2002;34:1996-2001.

9. Malina RM. Tracking of physical activity across the lifespan. Research Digest President's Council on Physical Fitness and Sports Series 3 [Internet]. 2001; 14:[3–10 pp.].

10. Telama R, Yang XL, Leskinen E, et al. Tracking of Physical Activity from Early Childhood through Youth into Adulthood. Med Sci Sport Exer 2014;46:955-62.

For peer review only

11. Telama R, Yang XL, Viikari J, et al. Physical activity from childhood to adulthood - A 21-year tracking study. Am J Prev Med 2005;28:267-73.

12. Smith L, Gardner B, Aggio D, et al. Association between participation in outdoor play and sport at 10 years old with physical activity in adulthood. Prev Med 2015;74:31-5.

13. Armstrong GK, Morgan K. Stability and change in levels of habitual physical activity in later life. Age Ageing 1998;27:17-23.

14. Morseth B, Jorgensen L, Emaus N, et al. Tracking of Leisure Time Physical Activity during 28 yr in Adults: The Tromso Study. Med Sci Sport Exer 2011;43:1229-34.

15. Dohle S, Wansink B. Fit in 50 years: participation in high school sports best predicts one's physical activity after Age 70. Bmc Public Health 2013;13.

16. Lennon LT, Ramsay SE, Papacosta O, et al. Cohort Profile Update: The British Regional Heart Study 1978-2014: 35 years follow-up of cardiovascular disease and ageing. Int J Epidemiol 2015;44.

17. Wilson PW, Paffenbarger RS, Jr., Morris JN, et al. Assessment methods for physical activity and physical fitness in population studies: report of a NHLBI workshop. Am Heart J 1986;111:1177-92.

18. Taylor HL, Jacobs DR, Jr., Schucker B, et al. A questionnaire for the assessment of leisure time physical activities. J Chronic Dis 1978;31:741-55.

19. Shaper AG, Wannamethee G, Weatherall R. Physical activity and ischaemic heart disease in middle-aged British men. Br Heart J 1991;66:384-94.

20. Jefferis BJ, Sartini C, Ash S, et al. Validity of questionnaire-based assessment of sedentary behaviour and physical activity in a population-based cohort of older men; comparisons with objectively measured physical activity data. Int J Behav Nutr Phy 2016;13.

21. Pocock SJ, Shaper AG, Cook DG, et al. Social class differences in ischaemic heart disease in British men. Lancet 1987;2:197-201.

For peer review only

22. Munoz SR, Bangdiwala SI. Interpretation of Kappa and B statistics measures of agreement. J Appl Stat 1997;24:105-11.

23. Smith L, Gardner B, Fisher A, et al. Patterns and correlates of physical activity behaviour over 10 years in older adults: prospective analyses from the English Longitudinal Study of Ageing. Bmj Open 2015;5.

24. Hamer M, Kivimaki M, Steptoe A. Longitudinal patterns in physical activity and sedentary behaviour from mid-life to early old age: a substudy of the Whitehall II cohort. J Epidemiol Commun H 2012;66:1110-5.

25. Cleland V, Dwyer T, Venn A. Which domains of childhood physical activity predict physical activity in adulthood? A 20-year prospective tracking study. Brit J Sport Med 2012;46:595-602.

For peer review only

Supplementary table 1. Stability of physical activity variables over time by changes in working status (n=3288)

	Wave 1 to 2	Wave 1 to 3	Wave 1 to 4
	Kappa	Kappa	Kappa
**Total Physical Activity**			
No change in working status	0.28	0.18	0.24
Retired between follow ups	0.25	0.25	0.25
**Sport participation**			
No change in working status	0.36	0.32	0.33
Retired between follow ups	0.40	0.35	0.35
**Recreational activity**			
No change in working status	0.26	0.18	0.27
Retired between follow ups	0.22	0.19	0.14
**Walking**			
No change in working status	0.18	0.16	0.13
Retired between follow ups	0.12	0.09	0.11

Note. Kappa statistics are presented for participants with a valid physical activity score at all four time points and valid data on working status n=3288. 46.4% (n=1526) of men retired between wave 1 and 2; 71.5% (n=2352) of men retired between wave 1 and 3; and 79.4% (n=2611) were retired between wave 1 and 4.

For peer review only

STROBE Statement—Checklist of items that should be included in reports of ***cohort studies***

	Item No	Recommendation
**Title and abstract**	1	(*a*) Indicate the study's design with a commonly used term in the title or the abstract. Page 2
		(*b*) Provide in the abstract an informative and balanced summary of what was done and what was found. Page 2
**Introduction**		
Background/rationale	2	Explain the scientific background and rationale for the investigation being reported. Page 4 to 5.
Objectives	3	State specific objectives, including any prespecified hypotheses. Page 5
**Methods**		
Study design	4	Present key elements of study design early in the paper. Page 5
Setting	5	Describe the setting, locations, and relevant dates, including periods of recruitment, exposure, follow-up, and data collection. Page 5
Participants	6	(*a*) Give the eligibility criteria, and the sources and methods of selection of participants. Describe methods of follow-up. Page 5
		(*b*) For matched studies, give matching criteria and number of exposed and unexposed. N/A
Variables	7	Clearly define all outcomes, exposures, predictors, potential confounders, and effect modifiers. Give diagnostic criteria, if applicable. Page 6 to 7
Data sources/ measurement	8*	For each variable of interest, give sources of data and details of methods of assessment (measurement). Describe comparability of assessment methods if there is more than one group. Page 6
Bias	9	Describe any efforts to address potential sources of bias. Page 7 to 8
Study size	10	Explain how the study size was arrived at. Page 8.
Quantitative variables	11	Explain how quantitative variables were handled in the analyses. If applicable, describe which groupings were chosen and why. Page 6 to 8.
Statistical methods	12	(*a*) Describe all statistical methods, including those used to control for confounding. Page 7 to 8
		(*b*) Describe any methods used to examine subgroups and interactions. Page 7 to 8
		(*c*) Explain how missing data were addressed. Page 7 to 8
		(*d*) If applicable, explain how loss to follow-up was addressed. Page 7 to 8
		(*e*) Describe any sensitivity analyses. Page 7 to 8.
**Results**		
Participants	13*	(a) Report numbers of individuals at each stage of study—eg numbers potentially eligible, examined for eligibility, confirmed eligible, included in the study, completing follow-up, and analysed. Page 8
		(b) Give reasons for non-participation at each stage. Page 8
		(c) Consider use of a flow diagram. N/A
Descriptive data	14*	(a) Give characteristics of study participants (eg demographic, clinical, social) and information on exposures and potential confounders. Page 9
		(b) Indicate number of participants with missing data for each variable of interest. Page 9 to 15.
		(c) Summarise follow-up time (eg, average and total amount). Page 5 and throughout
Outcome data	15*	Report numbers of outcome events or summary measures over time. Page 9
Main results	16	(*a*) Give unadjusted estimates and, if applicable, confounder-adjusted estimates and their precision (eg, 95% confidence interval). Make clear which confounders were

For peer review only - http://bmjopen.bmj.com/site/about/guidelines.xhtml

For peer review only

		adjusted for and why they were included. Page 13 to 15
		(*b*) Report category boundaries when continuous variables were categorized. Page 6
		(*c*) If relevant, consider translating estimates of relative risk into absolute risk for a meaningful time period. N/A
Other analyses	17	Report other analyses done—eg analyses of subgroups and interactions, and sensitivity analyses. See supplementary table
**Discussion**		
Key results	18	Summarise key results with reference to study objectives. Page 15 to 17
Limitations	19	Discuss limitations of the study, taking into account sources of potential bias or imprecision. Discuss both direction and magnitude of any potential bias. Page 18
Interpretation	20	Give a cautious overall interpretation of results considering objectives, limitations, multiplicity of analyses, results from similar studies, and other relevant evidence. Page 18
Generalisability	21	Discuss the generalisability (external validity) of the study results. Page 18
**Other information**		
Funding	22	Give the source of funding and the role of the funders for the present study and, if applicable, for the original study on which the present article is based. Page 19

*Give information separately for exposed and unexposed groups.

Note: An Explanation and Elaboration article discusses each checklist item and gives methodological background and published examples of transparent reporting. The STROBE checklist is best used in conjunction with this article (freely available on the Web sites of PLoS Medicine at http://www.plosmedicine.org/, Annals of Internal Medicine at http://www.annals.org/, and Epidemiology at http://www.epidem.com/). Information on the STROBE Initiative is available at http://www.strobe-statement.org.

For peer review only

Association between physical activity levels in midlife with physical activity in old age: A 20-year tracking study in a prospective cohort

Field	Value
Journal:	*BMJ Open*
Manuscript ID	bmjopen-2017-017378.R1
Article Type:	Research
Date Submitted by the Author:	29-Jun-2017
Complete List of Authors:	Aggio, Daniel; University College London, Department of Primary Care and Population Health<br>Papacosta, Olia; Department of Primary Care and Population Health London, UK<br>Lennon, Lucy; UCL, Department of Primary Care and Population Health<br>Whincup, Peter; St Georges, University of London, Population Health Research Institute<br>Wannamethee, Goya; University College London, Department of Primary Care and Population Health<br>Jefferis, Barbara ; University College London, Department of Primary Care and Population Health
**Primary Subject Heading**:	Epidemiology
Secondary Subject Heading:	Public health, Sports and exercise medicine
Keywords:	EPIDEMIOLOGY, PREVENTIVE MEDICINE, PUBLIC HEALTH

For peer review only

Association between physical activity levels in midlife with physical activity in old age: A 20-year tracking study in a prospective cohort

Aggio, Daniel[1,2]; Papacosta, Olia[1]; Lennon, Lucy T.[1]; Whincup, Peter H.[3]; Wannamethee S. Goya[1;] Jefferis, Barbara J.[1,2]

[1.] UCL Department of Primary Care & Population Health, UCL Medical School, London, United Kingdom

[2.] UCL PA Research Group, London, United Kingdom

[3.] Population Health Research Institute, St George's University of London, Cranmer Terrace, London, United Kingdom

Corresponding author: Daniel Aggio, UCL Department of Primary Care & Population Health, UCL Medical School, Rowland Hill Street, London NW3 2PF, UK.

Email d.aggio@ucl.ac.uk

Tel +44 207 830 2335

Fax: +44 20 7472 6871

Word count: 3920, 4 tables and 1 supplementary table

Keywords: aging, sport, physical activity

For peer review only

ABSTRACT

Objectives:

This study aims to examine the tracking and predictability of physical activity in old age from overall physical activity and participation in sport, recreational activity and walking in midlife.

Design:

Prospective population-based cohort study.

Setting:

British Regional Heart Study participants recruited from Primary Care Centres in the United Kingdom in 1978-80.

Participants and outcome measures:

Men (n=3413) self-reported their physical activity at baseline, 12-, 16- and 20-year follow ups and were categorised as inactive or active and having high or low participation in sport, walking and recreational activities. Tracking was assessed using kappa statistics and random effects models. Logistic regression estimated the odds of being active at 20-year follow up according to physical activity participation in midlife.

Results:

Among 3413 men (mean age at baseline 48.6 ± 5.4 years) with complete data, tracking of overall physical activity was moderate (kappa: 0.23-0.26). Tracking was higher for sports participation (kappa: 0.35-0.38) compared to recreational activity (kappa: 0.16-0.24) and walking (kappa: 0.11-0.15). Intraclass correlation coefficients (ICCs) demonstrated similar

For peer review only

levels of stability and only marginally weakened after controlling for covariates. Compared to inactive men, being active at baseline was associated with greater odds of being active at 20-year follow up (odds ratio (OR) 2.7, 95% confidence interval (CI), 2.4, 3.2) after adjusting for sociodemographic, health and lifestyle variables. Playing sport in midlife was more strongly associated with being active at 20-year follow up than other domains, particularly when sport participation begun earlier in life.

Conclusion:

Being physically active in midlife increases the odds of being active in old age. Promoting physical activity in later life might be best achieved by promoting sport participation earlier in the lifecourse.

Strengths and limitations of this study

- This study investigates the tracking of overall and specific domains of physical activity during the transition to old age over 20 years, an understudied period of the lifecourse.
- Very few studies have investigated the tracking of specific domains of physical activity during this period
- Our results may not be generalizable to women and non-white ethnic groups

For peer review only

Introduction

Prospective epidemiological studies have shown that physical activity (PA) in midlife and old age is associated with numerous health benefits, including reductions in cardiovascular disease (CVD) events and mortality [1, 2, 3]. Taking up PA in later life may reduce the risk of adverse health outcomes, but maintaining a physically active lifestyle throughout the life course may provide optimal health benefits [4, 5, 6]. The transition from midlife to old age typically coincides with major life events (e.g. retirement) and therefore may be an important window when both the volume and type of PA are likely to change. Knowledge on the stability, or tracking, of PA during this transition is very limited. The tracking of a behaviour over time can be determined by 1) its stability of over time, typically estimated using correlations between repeated measures or 2) the predictability of later measures from previous ones [7]. Past exercise behaviour is a consistent predictor of current PA levels [8]; however, few studies have examined the predictability of PA in old age from PA measures in midlife. Understanding tracking of PA during this transition may help inform interventions aiming to promote or maintain activity levels from midlife to old age.

There is a large body of research on the tracking of PA from childhood, but few studies have extended over prolonged periods in adults[9]. Current evidence suggests low to moderate tracking of PA throughout the lifecourse [9, 10, 11]. Studies tracking physical activity in youth have shown that sport participation in early life tracks more strongly [9] and is a stronger predictor of activity levels in adulthood (age 42 years) compared to other domains of activity such as outdoor play [12]. However, tracking studies in adults have rarely distinguished between the type of physical activity. Thus, it remains unknown what types of activity in midlife are more likely to predict PA in old age. The limited evidence in older adults has

For peer review only

suggested some domains of PA are more liable to change (e.g. indoor activities) than others (e.g. outdoor and leisure activities) [13] and thus may be easier to modify. Further studies have investigated the predictability of activity levels in early old age according to PA in early adulthood. For example, one study showed that being moderately active in young adulthood (mean age 35 years) increased the odds of being active 28 years later by more than three times [14]. Another study showed that sport participation in healthy young men (aged 25 years) strongly predicted PA 50 years later [15]. However, this study was retrospective in nature and may not be generalizable to less healthy populations.

Overall, very few tracking studies have extended into old age. Furthermore, the predictability of PA in later life from participation in specific types of activity in midlife remains unknown. Thus we aimed to estimate the tracking of overall and specific domains of PA from midlife to old age and the predictability of PA levels in old age from 1) overall PA and 2) PA domains in midlife.

Methods

Participants

Data were drawn from the British Regional Heart Study an ongoing prospective cohort study involving 7735 men (response rate = 78%) from 24 towns in Great Britain [16]. Men were recruited from primary care practices and were first examined in 1978-80 aged 40-59 years and were followed up after 12, 16 and 20 years. Response rates for surviving cohort members were 91% (n=5925), 88% (n=5263) and 77% (n=4252) at 12-, 16- and 20-year follow ups, respectively. Men completed a lifestyle and medical history questionnaire at the time of the examination (baseline and 20-year follow up) or by post (12- and 16-year follow ups).

For peer review only

Participants provided informed written consent to the investigation. Ethical approval was obtained from the National Research Ethics Service (NRES) Committee London.

Measures

Self-reported PA

At all waves, participants reported their usual PA levels. Questions referred to time spent on all forms of walking, time spent on recreational activities (such as recreational walking, gardening, chores, do-it-yourself (DIY) and how frequently they participate in sport/exercise. Responses to each domain of PA were scored based on the intensity and frequency of the activity [17, 18]. For example, making no journeys by foot was scored as 0 and >90 minutes/weekday was scored as 5. Scores were also heavily weighted for vigorous activities. For example, playing sport 4-7 times a month was given a score of 8. Scores for each domain were summed together to give a total PA score. The original scoring system has been reported in detail elsewhere [19]. The total PA score was then used to classify activity levels as inactive, occasional, light, moderate, moderately vigorous or vigorous. These PA scores have previously been validated against heart rate, forced expiratory volume in 1 second (FEV1) [19] and accelerometer-measured PA [20]. Results from the validation studies revealed a strong inverse relationship between PA and heart rate and a strong positive association with FEV1 and accelerometer-measured moderate-to-vigorous PA ($r=0.49$, $p < 0.001$)[19, 20]. For the purposes of this study the categories were grouped into active or inactive (inactive and occasional groups were classified as inactive). Responses to individual questions were also used to classify participation in specific domains of activity. Men were classified as having high or low sport/exercise participation (no sport/exercise participation was classified as low), high or low walking (low walking was classified as ≤20 mins/day) and high or low

For peer review only - http://bmjopen.bmj.com/site/about/guidelines.xhtml

recreational activity (low recreational activity was defined as being similar or less active than someone who spends two hours on most days on recreational activities). Men who reported participating in sport also retrospectively disclosed how many years they had been involved in that activity, from which men were classified as participating in sport for ≤ 4 years, 5-11 years, 12-24 years and ≥ 25 years.

Covariates

Participants self-reported their age at baseline; social class, which was derived from their longest held occupation [21] and categorised as manual or non-manual; and cigarette smoking habits, classified as current or ex-smokers and never smokers. Nurses measured participant's height and weight, which was used to derive body mass index. Men were then categorised as overweight or obese (Body Mass Index [BMI]: ≥25.0 Kg/m2) or healthy weight (BMI:<25.0 Kg/m2).

Statistical analysis

Descriptive statistics were used to report sample characteristics at baseline and the proportion of men active/high participation at each wave. McNemar's chi squared test was used to determine whether the proportion reporting being physically active changed between time points. Cohen's kappa was used to assess the observed agreement compared with the expected agreement. We followed suggestions by Munoz and Bangdiwala for interpretation of K coefficients : <0.00 indicates poor agreement, 0.00-0.20 fair agreement, 0.21-0.45 moderate agreement, 0.46-0.75 substantial agreement and 0.76-1.0 indicates near perfect agreement [22]. Random effects models were also used to calculate intraclass correlation

For peer review only

coefficients (ICCs), providing an indicator of tracking using data from all assessments whilst also controlling for covariates. In a supplementary analysis, we stratified our sample according to changes in employment status as we hypothesised that the timing of retirement may affect the stability of PA. We categorised men as no change in employment status (representing continuous employment/seeking employment and continuously retired) or retiring (i.e. retired between baseline and the respective follow up) and presented kappa statistics separately. Finally, we used logistic regression to estimate the odds ratio for being active compared to being inactive at 20-year follow up according to 1) overall activity levels at baseline, 2) engagement in specific domains of PA at baseline and 3) duration of sports participation. Tests for linear trend were also conducted by entering sports duration as a continuous variable into regression models. Initial models were adjusted for age, entered as a continuous variable (model 1) and then for BMI, social class and smoking status (categorical) (model 2). In analyses using just baseline activity levels as predictors of activity (i.e. not sports duration) at 20-year follow up, we also introduced a third model including all PA variables to identify the strongest predictor of activity 20 years later whilst also accounting for participation in other types of PA.

Results

7735 men responded to the baseline survey. Men who died during follow up (26.4%, n=2041), those with missing PA data for other reasons (29.4%, n=2272) at one or more examination between baseline and 20-year follow up and those with missing covariate data (0.1%, n=9) were excluded from analyses, leaving 3413 for analyses. Compared to men in the analytic sample, men excluded from the analyses were significantly older (baseline age, 48.6 vs. 51.5 years, p<0.001), had a higher BMI (baseline BMI, 25.3 vs. 25.7, p<0.001) and

For peer review only

were more likely to be inactive at baseline (proportion inactive at baseline, 55.5% vs. 66.1%, p<0.001). A larger sample was included in the random effects models, as men were only excluded if they did not provide PA measures on at least two assessments and have valid covariate data.

Table 1 displays sample characteristics and the proportion of men who were physically active and who participated in PA domains at each time point. Between baseline and 12-year follow up the number of men classified as active increased from 66.1% to 71.0% (p<0.001) and then dropped significantly to 63.7% and 66.9% (p<0.001) at 16- and 20-year follow ups, respectively. The proportion of men reporting high levels of walking increased from 26.9% at baseline to 61.5% at 20-year follow up (p<0.001). There were also steep declines over the 20-year follow up in recreational activity, with 56.0% of men reporting high levels of recreational activity at baseline and 40.2% at 20-year follow up.

Table 1. Sample characteristics and physical activity levels at baseline, 12-, 16- and 20-year follow up, n=3413

	Baseline	12 year	16 year	20 year
Age (years, mean ± SD)	48.6 ± 5.4	62.2 ± 5.4	66.2 ± 5.4	68.5 ± 5.4
Overweight/Obese (%, n)†	52.2 (1783)			
Current smoker (%, n)†	30.6 (1043)			
Manual Occupation (%, n)†	50.2 (1713)			
Physically active[a] (%, n)	66.1 (2257)	71.0 (2422)	63.7 (2173)	66.9 (2284)
High sport participation[b] (%, n)	47.7 (1627)	45.3 (1532)	44.6 (1493)	49.2 (1663)
High recreational activity[c] (%, n)	56.0 (1912)	58.4 (1994)	41.2 (1407)	40.2 (1372)
High walking[d] (%, n)	26.9 (918)	51.6 (1754)	50.9 (1735)	61.5 (2097)

Note. Data presented are for participants with a valid physical activity score at all four time points (n=3413). Data on walking was missing for an additional 15 participants at 12 year follow up, 3 participants at 16-year follow up and 1 participant at 20-year follow up. Data on sport participation was missing for 33 participants at 12 year follow up, 68 participants at 16 year follow up and 34 participants at 20 year follow up.

[a] Physically active was classified as reporting at least light activity.

For peer review only

[b] High sport was classified as reporting at least occasional participation (less than once a month)

[c] high recreational activity was classified as >2hours/day on recreational activities

[d] high walking was classified as >20 mins/day

†Data on BMI, smoking status and occupational class were utilised at baseline only

For peer review only

Table 2 presents kappa statistics and ICC for PA variables. Kappa statistics for overall PA ranged from 0.23-0.26 between baseline and subsequent time points, but were highest for sports participation (0.35-0.38) and lowest for walking (0.11-0.15). Kappa statistics were generally higher for shorter follow up periods. In random effects models, ICCs were consistent with the Kappa statistics and were only marginally weakened after controlling for covariates. In a supplementary analysis, we present Kappa statistics according to employment status. Overall stability of total PA was similar between men who reported no change in working status and men who retired between baseline and subsequent follow ups (see Supplementary Table 1). However, a higher proportion of men who were retiring increased their total activity between baseline and subsequent follow ups compared to men who reported no change in their working status (e.g. 21.3% vs. 15.7% of men increased their total activity levels between Wave 1 and Wave 2 in the retiring group and the no change group, respectively [data not shown]). Similarly, the overall stability of sport participation was comparable between men who reported no change in working status and retiring men, but the retiring group contained a higher proportion of men who increased their sport participation (e.g. 15.8% vs. 12.6% of men increased their sports participation between Wave 1 and Wave 2 in the retiring group and the no change group, respectively [data not shown]). Stability of recreational activity was markedly lower in men retiring between baseline and wave 4 compared to men who reported no change in their working status during the same period. This was largely explained by a higher proportion of retiring men reporting a decrease in recreational activity compared to men reporting no change in work status (e.g. 30.4% vs. 24.7% of men reported a decrease in recreational activity between Wave 1 and Wave 4 in the retiring group and the no change group, respectively). There were also some clear differences in the stability of walking activity between men who reported no change in working status and retiring men. This was largely explained by a higher proportion of retiring men reporting

For peer review only

an increase in walking activity compared to men with no change in working status (e.g. 39.6% vs. 28.9% of men reported an increase in walking activity between Wave 1 and Wave 2 in the retiring group and the no change group, respectively).

For peer review only

35 Table 2. Stability of physical activity variables over time, n=3413

	Wave 1 to 2	Wave 1 to 3	Wave 1 to 4	Random Effects Models	
				Univariate	Multivariate[a]
	Kappa	Kappa	Kappa	ICC (95% CI)	ICC (95% CI)
Physically active	0.26	0.23	0.24	0.46 (0.43, 0.48)	0.44 (0.41, 0.46)
Sport participation	0.38	0.35	0.35	0.65 (0.63, 0.67)	0.61 (0.59, 0.63)
Recreational activity	0.24	0.19	0.16	0.38 (0.36, 0.40)	0.36 (0.34, 0.39)
Walking	0.15	0.11	0.12	0.32 (0.30, 0.35)	0.32 (0.30, 0.34)

36 *Note.* Kappa statistics are presented for participants with a valid physical activity score at all four time points (n=3413). Data on walking was
37 missing for and additional 15 participants at 12 year follow up, 3 participants at 16-year follow up and 1 participant at 20-year follow up. Data
38 on sport participation was missing for 33 participants at 12 year follow up, 68 participants at 16 year follow up and 34 participants at 20 year
39 follow up. Random Effects Models included men with at least two assessments for each domain accompanied by valid covariate data (Physical
40 activity: n= 5962; Sport participation: n= 6122; Recreational activity: n=6093; Walking: n=6040). ICC = Intraclass correlation coefficients from
41 random effects models

42 [a] adjusted for age, BMI, social class and smoking status at baseline

For peer review only

Compared to inactive men, being physically active at baseline was associated with greater odds (OR 2.7, 95% CI, 2.4, 3.2) of being physically active at 20-year follow up after adjusting for age, social class, BMI and smoking status at baseline (Table 3). Odds ratios for being active at 20-year follow up were similarly raised for men who played sport at baseline after adjustments (OR 2.7 95% CI, 2.3, 3.1). High participation in walking and recreational activity at baseline were also associated with greater odds of being active at 20-year follow up (OR: 1.4-1.6). In the final model including all PA domains, sport participation at baseline remained the strongest predictor of being active at 20-year follow up.

Table 3. Odds of being active at 20 year follow up according to activity levels at baseline, n=3413

		Model 1	Model 2	Model 3
	N	OR (95% CI)	OR (95% CI)	OR (95% CI)
Physical activity				
Inactive	1,156	1.0	1.0	–
Active	2,257	2.9 (2.5, 3.3)	2.7 (2.4, 3.2)	–
Sport				
Low	1,786	1.0	1.0	1.0
High	1,627	2.9 (2.5, 3.4)	2.8 (2.4, 3.3)	2.7 (2.3, 3.1)
Recreational activity				
Low	1,501	1.0	1.0	1.0
High	1,912	1.9 (1.6, 2.2)	1.8 (1.6, 2.1)	1.6 (1.4, 1.9)
Walking				
Low	2,495	1.0	1.0	1.0
High	918	1.5 (1.3, 1.8)	1.5 (1.3, 1.8)	1.4 (1.2, 1.7)

Model 1 adjusted for baseline age. Model 2 additionally adjusted for social class, BMI and smoking status at baseline. Model 3 mutually adjusted for all domains of activity respectively.

For peer review only

Table 4. Odds of being active at 20 year follow up according to duration of sport participation at baseline (n=2969)

		Model 1	Model 2
	N	OR (95% CI)	OR (95% CI)
Sports participation duration			
Not participating at baseline	1786	1.0	1.0
≤ 4 years	262	2.4 (1.8, 3.2)	2.3 (1.7, 3.2)
5-11 years	331	3.0 (2.2, 4.0)	2.9 (2.2, 3.9)
12-24 years	290	4.4 (3.1, 6.2)	4.3 (3.1, 6.0)
≥ 25 years	300	5.0 (3.6, 7.1)	4.8 (3.4, 6.8)
p value for trend[a]		<0.001	<0.001

Model 1 adjusted for baseline age. Model 2 additionally adjusted for social class, BMI and smoking status at baseline.

[a] Tests for linear trend were conducted by entering sports duration as a continuous variable into regression models

Table 4 shows the odds of being active at 20-year follow up according to duration of sports participation from baseline. This sample size was lower than the main analytic sample as 27.3% (n=444) of men who played sport did not report duration of participation. Longer duration of sports participation was associated with increased odds of being active at 20-year follow up. Compared to those who were not participating in sport at baseline, taking part in sport for 25 years or more was most strongly associated with being active at 20-year follow up (OR 4.8, 95% CI, 3.4, 6.8). However, even taking up sport recently (≤ 4 years) was associated with increased odds of being active at 20-year follow up (OR 2.3, 95% CI, 1.7, 3.2).

Discussion

This study investigated the tracking of overall and specific domains of PA from midlife to old age and the predictability of PA in old age from overall PA and participation in sport,

For peer review only

recreational activity and walking in midlife. Agreement between overall PA levels at baseline and subsequent measures at 12-, 16- and 20-year follow ups suggested moderate levels of tracking, with stronger tracking evident for sport participation compared to other domains. Importantly, however, prevalence of walking >20 mins/day increased from around a third to approximately two thirds by 20-year follow up. Comparisons with previous studies are challenging given the various measures, cut off points and time frames studied, but our findings appear to be in agreement with previous studies in similar age groups [9, 23, 24], but over a longer time-span. As expected, tracking coefficients tended to be higher for the shorter follow up periods.

This is the first study that we are aware of to examine tracking of specific domains of activity from midlife to old age and the predictability of PA in old age from PA domains in midlife. Sports participation was the most stable domain with moderate agreement between baseline and subsequent time points. Tracking was fair for walking owing to a high proportion increasing their walking activity (e.g. 39.6% of retired men increased from low to high walking between baseline and 12-year follow up). Tracking of recreational activity was fair to moderate despite a large proportion decreasing their recreational activity by 20-year follow up (e.g. 30.5% of retired men decreased from high to low recreational activity between baseline and 12-year follow up). Tracking indicators from random effects models provided comparable estimates of tracking, whilst using all available measurements and controlling for factors that may influence tracking. The differential changes over time between domains of activity may reflect the changing opportunities and functional abilities associated with ageing. Our supplementary analyses suggest that retirement may be a period when PA behaviours are more sensitive to change. Increased free time during retirement may possibly explain the observed steep increase in walking and lower levels of stability during this transition, whereas declines in physical function and onset of chronic disease may explain

For peer review only

reductions in the more strenuous activities related to recreational activity, such as DIY and gardening. Given that our measure of recreational activity consisted of several activities including recreational walking, the observed increases in walking may have masked an even steeper decline in other recreational activities.

PA during midlife was associated with increased odds of being active in old age. Comparable results were found in a study by Morseth et al., [14], who found that Norwegian men and women who were active in midlife (aged 20-54 at baseline) were 5 to 13 times more likely to remain non-sedentary 28 years later. Although similar, our findings come from a sample of older British men all of whom would have transitioned to old age over the 20-year period and would take part in very different activities to their Norwegian counterparts. Sport participation in midlife predicted PA in old age more strongly than walking and recreational activity. This is consistent with previous tracking studies from childhood to adulthood that have also shown that sporting activity tracks more strongly [9] and is a stronger predictor of PA later in life than other domains of activity [12, 25]; however, this is the first study that we are aware of to demonstrate similar findings during the transition to old age. There may be a number of reasons why participation in sport in midlife more strongly predicts activity in older age than other types of activity. One possibility is that people's enjoyment of sport may be more likely to persist into old age than preferences for other types of activity. Sport participation in midlife may help maintain physical function and PA self-efficacy in later life, increasing psychological and physical readiness for PA in old age. Stronger levels of tracking may also suggest that participation in sport is less modifiable than other domains. By contrast, lower levels of tracking for walking, predominantly caused by large increases in uptake, suggest that walking may be easier to adopt in older adults, particularly those with functional limitations. Thus, improving our understanding on how to promote this domain of PA is important for future research. We also found that earlier engagement in sport more

For peer review only

strongly predicted PA in old age. Engagement in sport by early adulthood may be crucial for establishing a lifelong habit for sport participation and for developing important motor skills. Thus earlier engagement may result in improved capability to maintain PA and sport in older age. Although earlier sport participation appears favourable, even taking up sport in midlife more than doubled the odds of being active in old age compared to adults not taking part in sport in midlife. Encouraging sport participation early in the lifecourse may be most effective for promoting life-long PA but even interventions promoting uptake in middle-aged adults may be successful.

The main strengths of this study are its large sample size and long follow up, encompassing the transition from midlife to old age, an understudied period in PA tracking studies. Our study is limited by the use of self-reported assessment of PA, which may have been prone to bias. Nevertheless, the questionnaire was validated at baseline against heart rate and FEV1 [19] and more recently against accelerometer-measured PA [20]. Despite this, we are unable to validate responses to the question on duration of sport participation, although studies in men of a comparable age have used similar questions with longer recall periods.[15] Use of the same questionnaire at successive waves should result in comparable results between waves. Self-reports also allowed us to examine how specific types of PA track, which may provide useful insight for intervention strategy. Another limitation is that our findings may not be generalizable to women and non-white ethnic groups, who are not represented in this study sample. Furthermore, men who were lost to follow up were more likely to be overweight or obese and were generally less active than men with complete data. This attrition may have led to an overestimation of physical activity levels and the strength of tracking. Physical activity may be more liable to change in men who were lost to follow up, possibly as a result of an increased risk of developing chronic health conditions [26]. Random effects models which provide estimates of tracking using all available data, whilst also accounting for factors that

For peer review only

may influence tracking strength, may have alleviated, at least in part, the bias caused by this attrition.

Conclusion

In conclusion, PA tracks moderately from midlife to old age. Being active in midlife was associated with increased odds of being active in old age. Playing sport in midlife was more strongly associated with PA in old age than other domains of PA. Encouraging early and sustained sports participation into midlife may be effective for promoting PA in old age, but increased opportunities to take up other forms of activity, such as walking, may also be crucial as people transition into old age.

Footnotes

Contributors: SGW and PW designed and conceived the study. DA analysed and interpreted the data and drafted the initial manuscript. LL collected the data. OP generated the database. BJJ SGW interpreted the data and revised the manuscript. OP SGW PW LL BJJ DA approved the final manuscript.

Funding: DA is funded by a British Heart Foundation PhD studentship. This research was also supported by an NIHR Post-Doctoral Fellowship awarded to BJJ (2010–03–023) and by a British Heart Foundation project grant (PG/13/86/30546) to BJJ. The British Regional Heart study is supported by a British Heart Foundation grant (RG/13/16/30528).

Competing interests: None declared.

For peer review only

Ethical approval: Participants provided informed written consent to the investigation. Ethical approval was obtained from the National Research Ethics Service (NRES) Committee London.

Provenance and peer review: Not commissioned; externally peer reviewed.

Data sharing statement: Data are not publically available, but applications for data sharing can be made. For enquiries please contact Lucy Lennon (l.lennon@ucl.ac.uk).

For peer review only

References

1. Wannamethee SG, Shaper AG, Walker M, et al. Lifestyle and 15-year survival free of heart attack, stroke, and diabetes in middle-aged British men. Arch Intern Med 1998;158:2433-40.

2. Nocon M, Hiemann T, Muller-Riemenschneider F, et al. Association of physical activity with all-cause and cardiovascular mortality: a systematic review and meta-analysis. Eur J Cardiovasc Prev Rehabil 2008;15:239-46.

3. Li J, Siegrist J. Physical activity and risk of cardiovascular disease--a meta-analysis of prospective cohort studies. Int J Environ Res Public Health 2012;9:391-407.

4. Wannamethee SG, Shaper AG, Walker M. Changes in physical activity, mortality, and incidence of coronary heart disease in older men. Lancet 1998;351:1603-8.

5. Stessman J, Hammerman-Rozenberg R, Cohen A, et al. Physical Activity, Function, and Longevity Among the Very Old. Archives of Internal Medicine 2009;169:1476-83.

6. Byberg L, Melhus H, Gedeborg R, et al. Total mortality after changes in leisure time physical activity in 50 year old men: 35 year follow-up of population based cohort (Reprinted from BMJ, vol 338, b688, 2009). Brit J Sport Med 2009;43:482-U25.

7. Twisk JWR. Applied Longitudinal Data Analysis for Epidemiology A Practical Guide: Cambridge University Press; 2003.

8. Trost SG, Owen N, Bauman AE, et al. Correlates of adults' participation in physical activity: review and update. Med Sci Sport Exer 2002;34:1996-2001.

9. Malina RM. Tracking of physical activity across the lifespan. Research Digest President's Council on Physical Fitness and Sports Series 3 [Internet]. 2001; 14:[3–10 pp.].

10. Telama R, Yang XL, Leskinen E, et al. Tracking of Physical Activity from Early Childhood through Youth into Adulthood. Med Sci Sport Exer 2014;46:955-62.

11. Telama R, Yang XL, Viikari J, et al. Physical activity from childhood to adulthood - A 21-year tracking study. Am J Prev Med 2005;28:267-73.

12. Smith L, Gardner B, Aggio D, et al. Association between participation in outdoor play and sport at 10 years old with physical activity in adulthood. Prev Med 2015;74:31-5.

13. Armstrong GK, Morgan K. Stability and change in levels of habitual physical activity in later life. Age Ageing 1998;27:17-23.

14. Morseth B, Jorgensen L, Emaus N, et al. Tracking of Leisure Time Physical Activity during 28 yr in Adults: The Tromso Study. Med Sci Sport Exer 2011;43:1229-34.

15. Dohle S, Wansink B. Fit in 50 years: participation in high school sports best predicts one's physical activity after Age 70. Bmc Public Health 2013;13.

16. Lennon LT, Ramsay SE, Papacosta O, et al. Cohort Profile Update: The British Regional Heart Study 1978-2014: 35 years follow-up of cardiovascular disease and ageing. Int J Epidemiol 2015;44.

For peer review only

17. Wilson PW, Paffenbarger RS, Jr., Morris JN, et al. Assessment methods for physical activity and physical fitness in population studies: report of a NHLBI workshop. Am Heart J 1986;111:1177-92.

18. Taylor HL, Jacobs DR, Jr., Schucker B, et al. A questionnaire for the assessment of leisure time physical activities. J Chronic Dis 1978;31:741-55.

19. Shaper AG, Wannamethee G, Weatherall R. Physical activity and ischaemic heart disease in middle-aged British men. Br Heart J 1991;66:384-94.

20. Jefferis BJ, Sartini C, Ash S, et al. Validity of questionnaire-based assessment of sedentary behaviour and physical activity in a population-based cohort of older men; comparisons with objectively measured physical activity data. Int J Behav Nutr Phys Act 2016;13:14.

21. Pocock SJ, Shaper AG, Cook DG, et al. Social class differences in ischaemic heart disease in British men. Lancet 1987;2:197-201.

22. Munoz SR, Bangdiwala SI. Interpretation of Kappa and B statistics measures of agreement. J Appl Stat 1997;24:105-11.

23. Smith L, Gardner B, Fisher A, et al. Patterns and correlates of physical activity behaviour over 10 years in older adults: prospective analyses from the English Longitudinal Study of Ageing. Bmj Open 2015;5.

24. Hamer M, Kivimaki M, Steptoe A. Longitudinal patterns in physical activity and sedentary behaviour from mid-life to early old age: a substudy of the Whitehall II cohort. J Epidemiol Commun H 2012;66:1110-5.

25. Cleland V, Dwyer T, Venn A. Which domains of childhood physical activity predict physical activity in adulthood? A 20-year prospective tracking study. Brit J Sport Med 2012;46:595-602.

For peer review only

26. Walker M, Shaper AG, Cook DG. Non-participation and mortality in a prospective study of cardiovascular disease. J Epidemiol Community Health 1987;41:295-9.

For peer review only

Supplementary table 1. Stability of physical activity variables over time by changes in working status (n=3288)

	Wave 1 to 2	Wave 1 to 3	Wave 1 to 4
	Kappa	Kappa	Kappa
**Total Physical Activity**			
No change in working status	0.28	0.18	0.24
Retired between follow ups	0.25	0.25	0.25
**Sport participation**			
No change in working status	0.36	0.32	0.33
Retired between follow ups	0.40	0.35	0.35
**Recreational activity**			
No change in working status	0.26	0.18	0.27
Retired between follow ups	0.22	0.19	0.14
**Walking**			
No change in working status	0.18	0.16	0.13
Retired between follow ups	0.12	0.09	0.11

Note. Kappa statistics are presented for participants with a valid physical activity score at all four time points and valid data on working status n=3288. 46.4% (n=1526) of men retired between wave 1 and 2; 71.5% (n=2352) of men retired between wave 1 and 3; and 79.4% (n=2611) were retired between wave 1 and 4.

For peer review only

STROBE Statement—Checklist of items that should be included in reports of ***cohort studies***

	Item No	Recommendation
**Title and abstract**	1	(*a*) Indicate the study's design with a commonly used term in the title or the abstract<br>Page 1 - 2
		(*b*) Provide in the abstract an informative and balanced summary of what was done and what was found<br>Page 2-3
**Introduction**		
Background/rationale	2	Explain the scientific background and rationale for the investigation being reported<br>Page 4-5
Objectives	3	State specific objectives, including any prespecified hypotheses<br>Page 5
**Methods**		
Study design	4	Present key elements of study design early in the paper<br>Page 5
Setting	5	Describe the setting, locations, and relevant dates, including periods of recruitment, exposure, follow-up, and data collection<br>Page 5
Participants	6	(*a*) Give the eligibility criteria, and the sources and methods of selection of participants. Describe methods of follow-up<br>Page 5
		(*b*) For matched studies, give matching criteria and number of exposed and unexposed<br>N/A
Variables	7	Clearly define all outcomes, exposures, predictors, potential confounders, and effect modifiers. Give diagnostic criteria, if applicable<br>Page 6-7
Data sources/ measurement	8*	For each variable of interest, give sources of data and details of methods of assessment (measurement). Describe comparability of assessment methods if there is more than one group<br>Page 6-7
Bias	9	Describe any efforts to address potential sources of bias<br>Page 7-8
Study size	10	Explain how the study size was arrived at<br>Page 8-9
Quantitative variables	11	Explain how quantitative variables were handled in the analyses. If applicable, describe which groupings were chosen and why<br>Page 6-7
Statistical methods	12	(*a*) Describe all statistical methods, including those used to control for confounding<br>Page 7-8
		(*b*) Describe any methods used to examine subgroups and interactions<br>Page 8
		(*c*) Explain how missing data were addressed<br>Page 8
		(*d*) If applicable, explain how loss to follow-up was addressed<br>Page 8 and 18
		(*e*) Describe any sensitivity analyses<br>Page 8

For peer review only - http://bmjopen.bmj.com/site/about/guidelines.xhtml

**Results**		
Participants	13*	(a) Report numbers of individuals at each stage of study—eg numbers potentially eligible, examined for eligibility, confirmed eligible, included in the study, completing follow-up, and analysed<br>Page 8 and 12
		(b) Give reasons for non-participation at each stage<br>Page 8
		(c) Consider use of a flow diagram<br>N/A
Descriptive data	14*	(a) Give characteristics of study participants (eg demographic, clinical, social) and information on exposures and potential confounders<br>Table 1
		(b) Indicate number of participants with missing data for each variable of interest<br>See table footnotes
		(c) Summarise follow-up time (eg, average and total amount)<br>Page 5
Outcome data	15*	Report numbers of outcome events or summary measures over time<br>Page 5
Main results	16	(*a*) Give unadjusted estimates and, if applicable, confounder-adjusted estimates and their precision (eg, 95% confidence interval). Make clear which confounders were adjusted for and why they were included<br>Tables 3 and 4
		(*b*) Report category boundaries when continuous variables were categorized<br>Page 6-7
		(*c*) If relevant, consider translating estimates of relative risk into absolute risk for a meaningful time period<br>N/A
Other analyses	17	Report other analyses done—eg analyses of subgroups and interactions, and sensitivity analyses<br>Page 11
**Discussion**		
Key results	18	Summarise key results with reference to study objectives<br>Page 15-16
Limitations	19	Discuss limitations of the study, taking into account sources of potential bias or imprecision. Discuss both direction and magnitude of any potential bias<br>Page 18
Interpretation	20	Give a cautious overall interpretation of results considering objectives, limitations, multiplicity of analyses, results from similar studies, and other relevant evidence<br>Page 17-19
Generalisability	21	Discuss the generalisability (external validity) of the study results<br>Page 18
**Other information**		
Funding	22	Give the source of funding and the role of the funders for the present study and, if applicable, for the original study on which the present article is based<br>Page 19

*Give information separately for exposed and unexposed groups.

For peer review only - http://bmjopen.bmj.com/site/about/guidelines.xhtml

Note: An Explanation and Elaboration article discusses each checklist item and gives methodological background and published examples of transparent reporting. The STROBE checklist is best used in conjunction with this article (freely available on the Web sites of PLoS Medicine at http://www.plosmedicine.org/, Annals of Internal Medicine at http://www.annals.org/, and Epidemiology at http://www.epidem.com/). Information on the STROBE Initiative is available at http://www.strobe-statement.org.

For peer review only

For peer review only - http://bmjopen.bmj.com/site/about/guidelines.xhtml

BMJ Open

Association between physical activity levels in midlife with physical activity in old age: A 20-year tracking study in a prospective cohort

Journal:	*BMJ Open*
Manuscript ID	bmjopen-2017-017378.R2
Article Type:	Research
Date Submitted by the Author:	13-Jul-2017

Complete List of Authors:	Aggio, Daniel; University College London, Department of Primary Care and Population Health<br>Papacosta, Olia; Department of Primary Care and Population Health London, UK<br>Lennon, Lucy; UCL, Department of Primary Care and Population Health<br>Whincup, Peter; St Georges, University of London, Population Health Research Institute<br>Wannamethee, Goya; University College London, Department of Primary Care and Population Health<br>Jefferis, Barbara ; University College London, Department of Primary Care and Population Health

**Primary Subject Heading**:	Epidemiology
Secondary Subject Heading:	Public health, Sports and exercise medicine
Keywords:	EPIDEMIOLOGY, PREVENTIVE MEDICINE, PUBLIC HEALTH

SCHOLARONE™ Manuscripts

For peer review only

Association between physical activity levels in midlife with physical activity in old age: A 20-year tracking study in a prospective cohort

Aggio, Daniel[1,2]; Papacosta, Olia[1]; Lennon, Lucy T.[1]; Whincup, Peter H.[3]; Wannamethee S. Goya[1;] Jefferis, Barbara J.[1,2]

[1.] UCL Department of Primary Care & Population Health, UCL Medical School, London, United Kingdom

[2.] UCL PA Research Group, London, United Kingdom

[3.] Population Health Research Institute, St George's University of London, Cranmer Terrace, London, United Kingdom

Corresponding author: Daniel Aggio, UCL Department of Primary Care & Population Health, UCL Medical School, Rowland Hill Street, London NW3 2PF, UK.

Email d.aggio@ucl.ac.uk

Tel +44 207 830 2335

Fax: +44 20 7472 6871

Word count: 3920, 4 tables and 1 supplementary table

Keywords: aging, sport, physical activity

For peer review only

ABSTRACT

Objectives:

This study aims to examine the tracking and predictability of physical activity in old age from overall physical activity and participation in sport, recreational activity and walking in midlife.

Design:

Prospective population-based cohort study.

Setting:

British Regional Heart Study participants recruited from Primary Care Centres in the United Kingdom in 1978-80.

Participants and outcome measures:

Men (n=3413) self-reported their physical activity at baseline, 12-, 16- and 20-year follow ups and were categorised as inactive or active and having high or low participation in sport, walking and recreational activities. Tracking was assessed using kappa statistics and random effects models. Logistic regression estimated the odds of being active at 20-year follow up according to physical activity participation in midlife.

Results:

Among 3413 men (mean age at baseline 48.6 ± 5.4 years) with complete data, tracking of overall physical activity was moderate (kappa: 0.23-0.26). Tracking was higher for sports participation (kappa: 0.35-0.38) compared to recreational activity (kappa: 0.16-0.24) and walking (kappa: 0.11-0.15). Intraclass correlation coefficients (ICCs) demonstrated similar

For peer review only

levels of stability and only marginally weakened after controlling for covariates. Compared to inactive men, being active at baseline was associated with greater odds of being active at 20-year follow up (odds ratio (OR) 2.7, 95% confidence interval (CI), 2.4, 3.2) after adjusting for sociodemographic, health and lifestyle variables. Playing sport in midlife was more strongly associated with being active at 20-year follow up than other domains, particularly when sport participation begun earlier in life.

Conclusion:

Being physically active in midlife increases the odds of being active in old age. Promoting physical activity in later life might be best achieved by promoting sport participation earlier in the lifecourse.

Strengths and limitations of this study

- This study investigates the tracking of overall and specific domains of physical activity during the transition to old age over 20 years, an understudied period of the lifecourse.
- Very few studies have investigated the tracking of specific domains of physical activity during this period
- Our results may not be generalizable to women and non-white ethnic groups

For peer review only

Introduction

Prospective epidemiological studies have shown that physical activity (PA) in midlife and old age is associated with numerous health benefits, including reductions in cardiovascular disease (CVD) events and mortality [1, 2, 3]. Taking up PA in later life may reduce the risk of adverse health outcomes, but maintaining a physically active lifestyle throughout the life course may provide optimal health benefits [4, 5, 6]. The transition from midlife to old age typically coincides with major life events (e.g. retirement) and therefore may be an important window when both the volume and type of PA are likely to change. Knowledge on the stability, or tracking, of PA during this transition is very limited. The tracking of a behaviour over time can be determined by 1) its stability of over time, typically estimated using correlations between repeated measures or 2) the predictability of later measures from previous ones [7]. Past exercise behaviour is a consistent predictor of current PA levels [8]; however, few studies have examined the predictability of PA in old age from PA measures in midlife. Understanding tracking of PA during this transition may help inform interventions aiming to promote or maintain activity levels from midlife to old age.

There is a large body of research on the tracking of PA from childhood, but few studies have extended over prolonged periods in adults[9]. Current evidence suggests low to moderate tracking of PA throughout the lifecourse [9, 10, 11]. Studies tracking physical activity in youth have shown that sport participation in early life tracks more strongly [9] and is a stronger predictor of activity levels in adulthood (age 42 years) compared to other domains of activity such as outdoor play [12]. However, tracking studies in adults have rarely distinguished between the type of physical activity. Thus, it remains unknown what types of activity in midlife are more likely to predict PA in old age. The limited evidence in older adults has

For peer review only

suggested some domains of PA are more liable to change (e.g. indoor activities) than others (e.g. outdoor and leisure activities) [13] and thus may be easier to modify. Further studies have investigated the predictability of activity levels in early old age according to PA in early adulthood. For example, one study showed that being moderately active in young adulthood (mean age 35 years) increased the odds of being active 28 years later by more than three times [14]. Another study showed that sport participation in healthy young men (aged 25 years) strongly predicted PA 50 years later [15]. However, this study was retrospective in nature and may not be generalizable to less healthy populations.

Overall, very few tracking studies have extended into old age. Furthermore, the predictability of PA in later life from participation in specific types of activity in midlife remains unknown. Thus we aimed to estimate the tracking of overall and specific domains of PA from midlife to old age and the predictability of PA levels in old age from 1) overall PA and 2) PA domains in midlife.

Methods

Participants

Data were drawn from the British Regional Heart Study an ongoing prospective cohort study involving 7735 men (response rate = 78%) from 24 towns in Great Britain [16]. Men were recruited from primary care practices and were first examined in 1978-80 aged 40-59 years and were followed up after 12, 16 and 20 years. Response rates for surviving cohort members were 91% (n=5925), 88% (n=5263) and 77% (n=4252) at 12-, 16- and 20-year follow ups, respectively. Men completed a lifestyle and medical history questionnaire at the time of the examination (baseline and 20-year follow up) or by post (12- and 16-year follow ups).

For peer review only - http://bmjopen.bmj.com/site/about/guidelines.xhtml

Participants provided informed written consent to the investigation. Ethical approval was obtained from the National Research Ethics Service (NRES) Committee London.

Measures

Self-reported PA

At all waves, participants reported their usual PA levels. Questions referred to time spent on all forms of walking, time spent on recreational activities (such as recreational walking, gardening, chores, do-it-yourself (DIY) and how frequently they participate in sport/exercise. Responses to each domain of PA were scored based on the intensity and frequency of the activity [17, 18]. For example, making no journeys by foot was scored as 0 and >90 minutes/weekday was scored as 5. Scores were also heavily weighted for vigorous activities. For example, playing sport 4-7 times a month was given a score of 8. Scores for each domain were summed together to give a total PA score. The original scoring system has been reported in detail elsewhere [19]. The total PA score was then used to classify activity levels as inactive, occasional, light, moderate, moderately vigorous or vigorous. These PA scores have previously been validated against resting heart rate [19] and accelerometer-measured PA [20]. Results from the validation studies revealed a strong inverse relationship between PA and electrocardiogram-measured resting heart rate ($p<0.001$) and a strong positive association with accelerometer-measured moderate-to-vigorous PA ($r=0.49$, $p < 0.001$)[19, 20]. For the purposes of this study the categories were grouped into active or inactive (inactive and occasional groups were classified as inactive). Responses to individual questions were also used to classify participation in specific domains of activity. Men were classified as having high or low sport/exercise participation (no sport/exercise participation was classified as low), high or low walking (low walking was classified as ≤20 mins/day) and high or low

For peer review only - http://bmjopen.bmj.com/site/about/guidelines.xhtml

recreational activity (low recreational activity was defined as being similar or less active than someone who spends two hours on most days on recreational activities). Men who reported participating in sport also retrospectively disclosed how many years they had been involved in that activity, from which men were classified as participating in sport for ≤ 4 years, 5-11 years, 12-24 years and ≥ 25 years.

Covariates

Participants self-reported their age at baseline; social class, which was derived from their longest held occupation [21] and categorised as manual or non-manual; and cigarette smoking habits, classified as current or ex-smokers and never smokers. Nurses measured participant's height and weight, which was used to derive body mass index. Men were then categorised as overweight or obese (Body Mass Index [BMI]: ≥25.0 Kg/m2) or healthy weight (BMI:<25.0 Kg/m2).

Statistical analysis

Descriptive statistics were used to report sample characteristics at baseline and the proportion of men active/high participation at each wave. McNemar's chi squared test was used to determine whether the proportion reporting being physically active changed between time points. Cohen's kappa was used to assess the observed agreement compared with the expected agreement. We followed suggestions by Munoz and Bangdiwala for interpretation of K coefficients : <0.00 indicates poor agreement, 0.00-0.20 fair agreement, 0.21-0.45 moderate agreement, 0.46-0.75 substantial agreement and 0.76-1.0 indicates near perfect agreement [22]. Kappa statistics vary in magnitude depending on how the outcome measure is

For peer review only

categorised. To be consistent across all our measures we decided to perform analyses using binary variables. Random effects models were also used to calculate intraclass correlation coefficients (ICCs), providing an indicator of tracking using data from all assessments whilst also controlling for covariates. In a supplementary analysis, we stratified our sample according to changes in employment status as we hypothesised that the timing of retirement may affect the stability of PA. We categorised men as no change in employment status (representing continuous employment/seeking employment and continuously retired) or retiring (i.e. retired between baseline and the respective follow up) and presented kappa statistics separately. Finally, we used logistic regression to estimate the odds ratio for being active compared to being inactive at 20-year follow up according to 1) overall activity levels at baseline, 2) engagement in specific domains of PA at baseline and 3) duration of sports participation. Tests for linear trend were also conducted by entering sports duration as a continuous variable into regression models. Initial models were adjusted for age, entered as a continuous variable (model 1) and then for BMI, social class and smoking status (categorical) (model 2). In analyses using just baseline activity levels as predictors of activity (i.e. not sports duration) at 20-year follow up, we also introduced a third model including all PA variables to identify the strongest predictor of activity 20 years later whilst also accounting for participation in other types of PA.

For peer review only

Results

7735 men responded to the baseline survey. Men who died during follow up (26.4%, n=2041), those with missing PA data for other reasons (29.4%, n=2272) at one or more examination between baseline and 20-year follow up and those with missing covariate data (0.1%, n=9) were excluded from analyses, leaving 3413 for analyses. Compared to men in

the analytic sample, men excluded from the analyses were significantly older (baseline age, 48.6 vs. 51.5 years, p<0.001), had a higher BMI (baseline BMI, 25.3 vs. 25.7, p<0.001) and were more likely to be inactive at baseline (proportion inactive at baseline, 55.5% vs. 66.1%, p<0.001). A larger sample was included in the random effects models, as men were only excluded if they did not provide PA measures on at least two assessments and have valid covariate data.

Table 1 displays sample characteristics and the proportion of men who were physically active and who participated in PA domains at each time point. Between baseline and 12-year follow up the number of men classified as active increased from 66.1% to 71.0% (p<0.001) and then dropped significantly to 63.7% and 66.9% (p<0.001) at 16- and 20-year follow ups, respectively. The proportion of men classified as active declined more rapidly thereafter, with 57.3% of men classified as active at 30-year follow up (data not shown). The proportion of men reporting high levels of walking increased from 26.9% at baseline to 61.5% at 20-year follow up (p<0.001). There were also steep declines over the 20-year follow up in recreational activity, with 56.0% of men reporting high levels of recreational activity at baseline and 40.2% at 20-year follow up.

Table 1. Sample characteristics and physical activity levels at baseline, 12-, 16- and 20-year follow up, n=3413

	Baseline	12 year	16 year	20 year
Age (years, mean ± SD)	48.6 ± 5.4	62.2 ± 5.4	66.2 ± 5.4	68.5 ± 5.4
Overweight/Obese (%, n)†	52.2 (1783)			
Current smoker (%, n)†	30.6 (1043)			
Manual Occupation (%, n)†	50.2 (1713)			
Physically active[a] (%, n)	66.1 (2257)	71.0 (2422)	63.7 (2173)	66.9 (2284)
High sport participation[b] (%, n)	47.7 (1627)	45.3 (1532)	44.6 (1493)	49.2 (1663)
High recreational activity[c] (%, n)	56.0 (1912)	58.4 (1994)	41.2 (1407)	40.2 (1372)
High walking[d] (%, n)	26.9 (918)	51.6 (1754)	50.9 (1735)	61.5 (2097)

For peer review only

Note. Data presented are for participants with a valid physical activity score at all four time points (n=3413). Data on walking was missing for an additional 15 participants at 12 year follow up, 3 participants at 16-year follow up and 1 participant at 20-year follow up. Data on sport participation was missing for 33 participants at 12 year follow up, 68 participants at 16 year follow up and 34 participants at 20 year follow up.

[a] Physically active was classified as reporting at least light activity.

[b] High sport was classified as reporting at least occasional participation (less than once a month)

[c] high recreational activity was classified as >2hours/day on recreational activities

[d] high walking was classified as >20 mins/day

†Data on BMI, smoking status and occupational class were utilised at baseline only

For peer review only

Table 2 presents kappa statistics and ICC for PA variables. Kappa statistics for overall PA ranged from 0.23-0.26 between baseline and subsequent time points, but were highest for sports participation (0.35-0.38) and lowest for walking (0.11-0.15). Kappa statistics were generally higher for shorter follow up periods. In random effects models, ICCs were consistent with the Kappa statistics and were only marginally weakened after controlling for covariates. In a supplementary analysis, we present Kappa statistics according to employment status. Overall stability of total PA was similar between men who reported no change in working status and men who retired between baseline and subsequent follow ups (see Supplementary Table 1). However, a higher proportion of men who were retiring increased their total activity between baseline and subsequent follow ups compared to men who reported no change in their working status (e.g. 21.3% vs. 15.7% of men increased their total activity levels between Wave 1 and Wave 2 in the retiring group and the no change group, respectively [data not shown]). Similarly, the overall stability of sport participation was comparable between men who reported no change in working status and retiring men, but the retiring group contained a higher proportion of men who increased their sport participation (e.g. 15.8% vs. 12.6% of men increased their sports participation between Wave 1 and Wave 2 in the retiring group and the no change group, respectively [data not shown]). Stability of recreational activity was markedly lower in men retiring between baseline and wave 4 compared to men who reported no change in their working status during the same period. This was largely explained by a higher proportion of retiring men reporting a decrease in recreational activity compared to men reporting no change in work status (e.g. 30.4% vs. 24.7% of men reported a decrease in recreational activity between Wave 1 and Wave 4 in the retiring group and the no change group, respectively). There were also some clear differences in the stability of walking activity between men who reported no change in working status and retiring men. This was largely explained by a higher proportion of retiring men reporting

For peer review only

an increase in walking activity compared to men with no change in working status (e.g. 39.6% vs. 28.9% of men reported an increase in walking activity between Wave 1 and Wave 2 in the retiring group and the no change group, respectively).

For peer review only

Table 2. Stability of physical activity variables over time, n=3413

	Wave 1 to 2	Wave 1 to 3	Wave 1 to 4	Random Effects Models	
				Univariate	Multivariate[a]
	Kappa	Kappa	Kappa	ICC (95% CI)	ICC (95% CI)
Physically active	0.26	0.23	0.24	0.46 (0.43, 0.48)	0.44 (0.41, 0.46)
Sport participation	0.38	0.35	0.35	0.65 (0.63, 0.67)	0.61 (0.59, 0.63)
Recreational activity	0.24	0.19	0.16	0.38 (0.36, 0.40)	0.36 (0.34, 0.39)
Walking	0.15	0.11	0.12	0.32 (0.30, 0.35)	0.32 (0.30, 0.34)

Note. Kappa statistics are presented for participants with a valid physical activity score at all four time points (n=3413). Data on walking was missing for and additional 15 participants at 12 year follow up, 3 participants at 16-year follow up and 1 participant at 20-year follow up. Data on sport participation was missing for 33 participants at 12 year follow up, 68 participants at 16 year follow up and 34 participants at 20 year follow up. Random Effects Models included men with at least two assessments for each domain accompanied by valid covariate data (Physical activity: n= 5962; Sport participation: n= 6122; Recreational activity: n=6093; Walking: n=6040). ICC = Intraclass correlation coefficients from random effects models

[a] adjusted for age, BMI, social class and smoking status at baseline

For peer review only

Compared to inactive men, being physically active at baseline was associated with greater odds (OR 2.7, 95% CI, 2.4, 3.2) of being physically active at 20-year follow up after adjusting for age, social class, BMI and smoking status at baseline (Table 3). Odds ratios for being active at 20-year follow up were similarly raised for men who played sport at baseline after adjustments (OR 2.7 95% CI, 2.3, 3.1). High participation in walking and recreational activity at baseline were also associated with greater odds of being active at 20-year follow up (OR: 1.4-1.6). In the final model including all PA domains, sport participation at baseline remained the strongest predictor of being active at 20-year follow up.

Table 3. Odds of being active at 20 year follow up according to activity levels at baseline, n=3413

		Model 1	Model 2	Model 3
	N	OR (95% CI)	OR (95% CI)	OR (95% CI)
Physical activity				
Inactive	1,156	1.0	1.0	–
Active	2,257	2.9 (2.5, 3.3)	2.7 (2.4, 3.2)	–
Sport				
Low	1,786	1.0	1.0	1.0
High	1,627	2.9 (2.5, 3.4)	2.8 (2.4, 3.3)	2.7 (2.3, 3.1)
Recreational activity				
Low	1,501	1.0	1.0	1.0
High	1,912	1.9 (1.6, 2.2)	1.8 (1.6, 2.1)	1.6 (1.4, 1.9)
Walking				
Low	2,495	1.0	1.0	1.0
High	918	1.5 (1.3, 1.8)	1.5 (1.3, 1.8)	1.4 (1.2, 1.7)

Model 1 adjusted for baseline age. Model 2 additionally adjusted for social class, BMI and smoking status at baseline. Model 3 mutually adjusted for all domains of activity respectively.

For peer review only

Table 4. Odds of being active at 20 year follow up according to duration of sport participation at baseline (n=2969)

		Model 1	Model 2
	N	OR (95% CI)	OR (95% CI)
Sports participation duration			
Not participating at baseline	1786	1.0	1.0
≤ 4 years	262	2.4 (1.8, 3.2)	2.3 (1.7, 3.2)
5-11 years	331	3.0 (2.2, 4.0)	2.9 (2.2, 3.9)
12-24 years	290	4.4 (3.1, 6.2)	4.3 (3.1, 6.0)
≥ 25 years	300	5.0 (3.6, 7.1)	4.8 (3.4, 6.8)
p value for trend[a]		<0.001	<0.001

Model 1 adjusted for baseline age. Model 2 additionally adjusted for social class, BMI and smoking status at baseline.

[a] Tests for linear trend were conducted by entering sports duration as a continuous variable into regression models

Table 4 shows the odds of being active at 20-year follow up according to duration of sports participation from baseline. This sample size was lower than the main analytic sample as 27.3% (n=444) of men who played sport did not report duration of participation. Longer duration of sports participation was associated with increased odds of being active at 20-year follow up. Compared to those who were not participating in sport at baseline, taking part in sport for 25 years or more was most strongly associated with being active at 20-year follow up (OR 4.8, 95% CI, 3.4, 6.8). However, even taking up sport recently (≤ 4 years) was associated with increased odds of being active at 20-year follow up (OR 2.3, 95% CI, 1.7, 3.2).

Discussion

This study investigated the tracking of overall and specific domains of PA from midlife to old age and the predictability of PA in old age from overall PA and participation in sport,

For peer review

recreational activity and walking in midlife. Agreement between overall PA levels at baseline and subsequent measures at 12-, 16- and 20-year follow ups suggested moderate levels of tracking, with stronger tracking evident for sport participation compared to other domains. Importantly, however, prevalence of walking >20 mins/day increased from around a third to approximately two thirds by 20-year follow up. Comparisons with previous studies are challenging given the various measures, cut off points and time frames studied, but our findings appear to be in agreement with previous studies in similar age groups [9, 23, 24], but over a longer time-span. As expected, tracking coefficients tended to be higher for the shorter follow up periods. Although the proportion of men categorized as active fluctuated over the 20-year follow up period, we did not observe the decline over time that one might expect with the advancing age of the sample. However, when we extended our follow up to 30 years when men were aged 70-89, a notable decline was observed. This is consistent with cross-sectional data from the Health Survey for England [25], which presented similar proportions of men meeting physical activity recommendations at ages 55 to 64 and at ages 65 to 74 years, followed by a decline in men >75 years old.

This is the first study that we are aware of to examine tracking of specific domains of activity from midlife to old age and the predictability of PA in old age from PA domains in midlife. Sports participation was the most stable domain with moderate agreement between baseline and subsequent time points. Tracking was fair for walking owing to a high proportion increasing their walking activity (e.g. 39.6% of retired men increased from low to high walking between baseline and 12-year follow up). Tracking of recreational activity was fair to moderate despite a large proportion decreasing their recreational activity by 20-year follow up (e.g. 30.5% of retired men decreased from high to low recreational activity between baseline and 12-year follow up). Tracking indicators from random effects models provided comparable estimates of tracking, whilst using all available measurements and controlling for

For peer review only

108 factors that may influence tracking. The differential changes over time between domains of activity may reflect the changing opportunities and functional abilities associated with ageing. Our supplementary analyses suggest that retirement may be a period when PA behaviours are more sensitive to change. Increased free time during retirement may possibly explain the observed steep increase in walking and lower levels of stability during this transition, whereas declines in physical function and onset of chronic disease may explain reductions in the more strenuous activities related to recreational activity, such as DIY and gardening. Given that our measure of recreational activity consisted of several activities including recreational walking, the observed increases in walking may have masked an even steeper decline in other recreational activities.

118 PA during midlife was associated with increased odds of being active in old age. Comparable results were found in a study by Morseth et al., [14], who found that Norwegian men and women who were active in midlife (aged 20-54 at baseline) were 5 to 13 times more likely to remain non-sedentary 28 years later. Although similar, our findings come from a sample of older British men all of whom would have transitioned to old age over the 20-year period and would take part in very different activities to their Norwegian counterparts. Sport participation in midlife predicted PA in old age more strongly than walking and recreational activity. This is consistent with previous tracking studies from childhood to adulthood that have also shown that sporting activity tracks more strongly [9] and is a stronger predictor of PA later in life than other domains of activity [12, 26]; however, this is the first study that we are aware of to demonstrate similar findings during the transition to old age. There may be a number of reasons why participation in sport in midlife more strongly predicts activity in older age than other types of activity. One possibility is that people's enjoyment of sport may be more likely to persist into old age than preferences for other types of activity. Sport participation in midlife may help maintain physical function and PA self-efficacy in later life,

For peer review only

increasing psychological and physical readiness for PA in old age. Stronger levels of tracking may also suggest that participation in sport is less modifiable than other domains. By contrast, lower levels of tracking for walking, predominantly caused by large increases in uptake, suggest that walking may be easier to adopt in older adults, particularly those with functional limitations. Thus, improving our understanding on how to promote this domain of PA is important for future research. We also found that earlier engagement in sport more strongly predicted PA in old age. Engagement in sport by early adulthood may be crucial for establishing a lifelong habit for sport participation and for developing important motor skills. Thus earlier engagement may result in improved capability to maintain PA and sport in older age. Although earlier sport participation appears favourable, even taking up sport in midlife more than doubled the odds of being active in old age compared to adults not taking part in sport in midlife. Encouraging sport participation early in the lifecourse may be most effective for promoting life-long PA but even interventions promoting uptake in middle-aged adults may be successful.

The main strengths of this study are its large sample size and long follow up, encompassing the transition from midlife to old age, an understudied period in PA tracking studies. Our study is limited by the use of self-reported assessment of PA, which may have been prone to bias. Nevertheless, the questionnaire was validated at baseline against resting heart rate [19] and more recently against accelerometer-measured PA [20]. Despite this, we are unable to validate responses to the question on duration of sport participation, although studies in men of a comparable age have used similar questions with longer recall periods.[15] Use of the same questionnaire at successive waves should result in comparable results between waves. Self-reports also allowed us to examine how specific types of PA track, which may provide useful insight for intervention strategy. Another limitation is that our findings may not be generalizable to women and non-white ethnic groups, who are not represented in this study

For peer review only

sample. Furthermore, men who were lost to follow up were more likely to be overweight or obese and were generally less active than men with complete data. This attrition may have led to an overestimation of physical activity levels and the strength of tracking. Physical activity may be more liable to change in men who were lost to follow up, possibly as a result of an increased risk of developing chronic health conditions [27]. Random effects models which provide estimates of tracking using all available data, whilst also accounting for factors that may influence tracking strength, may have alleviated, at least in part, the bias caused by this attrition.

Conclusion

In conclusion, PA tracks moderately from midlife to old age. Being active in midlife was associated with increased odds of being active in old age. Playing sport in midlife was more strongly associated with PA in old age than other domains of PA. Encouraging early and sustained sports participation into midlife may be effective for promoting PA in old age, but increased opportunities to take up other forms of activity, such as walking, may also be crucial as people transition into old age.

Footnotes

Contributors: SGW and PW designed and conceived the study. DA analysed and interpreted the data and drafted the initial manuscript. LL collected the data. OP generated the database. BJJ SGW interpreted the data and revised the manuscript. OP SGW PW LL BJJ DA approved the final manuscript.

For peer review only

Funding: DA is funded by a British Heart Foundation PhD studentship. This research was also supported by an NIHR Post-Doctoral Fellowship awarded to BJJ (2010–03–023) and by a British Heart Foundation project grant (PG/13/86/30546) to BJJ. The British Regional Heart study is supported by a British Heart Foundation grant (RG/13/16/30528).

Competing interests: None declared.

Ethical approval: Participants provided informed written consent to the investigation. Ethical approval was obtained from the National Research Ethics Service (NRES) Committee London.

Provenance and peer review: Not commissioned; externally peer reviewed.

Data sharing statement: Data are not publically available, but applications for data sharing can be made. For enquiries please contact Lucy Lennon (l.lennon@ucl.ac.uk).

References

1. Wannamethee SG, Shaper AG, Walker M, et al. Lifestyle and 15-year survival free of heart attack, stroke, and diabetes in middle-aged British men. Arch Intern Med 1998;158:2433-40.
2. Nocon M, Hiemann T, Muller-Riemenschneider F, et al. Association of physical activity with all-cause and cardiovascular mortality: a systematic review and meta-analysis. Eur J Cardiovasc Prev Rehabil 2008;15:239-46.
3. Li J, Siegrist J. Physical activity and risk of cardiovascular disease--a meta-analysis of prospective cohort studies. Int J Environ Res Public Health 2012;9:391-407.

For peer review only

4. Wannamethee SG, Shaper AG, Walker M. Changes in physical activity, mortality, and incidence of coronary heart disease in older men. Lancet 1998;351:1603-8.

5. Stessman J, Hammerman-Rozenberg R, Cohen A, et al. Physical Activity, Function, and Longevity Among the Very Old. Archives of Internal Medicine 2009;169:1476-83.

6. Byberg L, Melhus H, Gedeborg R, et al. Total mortality after changes in leisure time physical activity in 50 year old men: 35 year follow-up of population based cohort (Reprinted from BMJ, vol 338, b688, 2009). Brit J Sport Med 2009;43:482-U25.

7. Twisk JWR. Applied Longitudinal Data Analysis for Epidemiology A Practical Guide: Cambridge University Press; 2003.

8. Trost SG, Owen N, Bauman AE, et al. Correlates of adults' participation in physical activity: review and update. Med Sci Sport Exer 2002;34:1996-2001.

9. Malina RM. Tracking of physical activity across the lifespan. Research Digest President's Council on Physical Fitness and Sports Series 3 [Internet]. 2001; 14:[3–10 pp.].

10. Telama R, Yang XL, Leskinen E, et al. Tracking of Physical Activity from Early Childhood through Youth into Adulthood. Med Sci Sport Exer 2014;46:955-62.

11. Telama R, Yang XL, Viikari J, et al. Physical activity from childhood to adulthood - A 21-year tracking study. Am J Prev Med 2005;28:267-73.

12. Smith L, Gardner B, Aggio D, et al. Association between participation in outdoor play and sport at 10 years old with physical activity in adulthood. Prev Med 2015;74:31-5.

13. Armstrong GK, Morgan K. Stability and change in levels of habitual physical activity in later life. Age Ageing 1998;27:17-23.

14. Morseth B, Jorgensen L, Emaus N, et al. Tracking of Leisure Time Physical Activity during 28 yr in Adults: The Tromso Study. Med Sci Sport Exer 2011;43:1229-34.

15. Dohle S, Wansink B. Fit in 50 years: participation in high school sports best predicts one's physical activity after Age 70. Bmc Public Health 2013;13.

16. Lennon LT, Ramsay SE, Papacosta O, et al. Cohort Profile Update: The British Regional Heart Study 1978-2014: 35 years follow-up of cardiovascular disease and ageing. Int J Epidemiol 2015;44.

For peer review only

17. Wilson PW, Paffenbarger RS, Jr., Morris JN, et al. Assessment methods for physical activity and physical fitness in population studies: report of a NHLBI workshop. Am Heart J 1986;111:1177-92.

18. Taylor HL, Jacobs DR, Jr., Schucker B, et al. A questionnaire for the assessment of leisure time physical activities. J Chronic Dis 1978;31:741-55.

19. Shaper AG, Wannamethee G, Weatherall R. Physical activity and ischaemic heart disease in middle-aged British men. Br Heart J 1991;66:384-94.

20. Jefferis BJ, Sartini C, Ash S, et al. Validity of questionnaire-based assessment of sedentary behaviour and physical activity in a population-based cohort of older men; comparisons with objectively measured physical activity data. Int J Behav Nutr Phys Act 2016;13:14.

21. Pocock SJ, Shaper AG, Cook DG, et al. Social class differences in ischaemic heart disease in British men. Lancet 1987;2:197-201.

22. Munoz SR, Bangdiwala SI. Interpretation of Kappa and B statistics measures of agreement. J Appl Stat 1997;24:105-11.

23. Smith L, Gardner B, Fisher A, et al. Patterns and correlates of physical activity behaviour over 10 years in older adults: prospective analyses from the English Longitudinal Study of Ageing. Bmj Open 2015;5.

24. Hamer M, Kivimaki M, Steptoe A. Longitudinal patterns in physical activity and sedentary behaviour from mid-life to early old age: a substudy of the Whitehall II cohort. J Epidemiol Commun H 2012;66:1110-5.

25. Scholes S, Mindell J. Physical activity in adults. In: Craig R, Mindell J, editors. Health Survey for England 2012: Health, social care and lifestyles. Volume 1. Leeds: The Health and Social Care Information Centre; 2014: p.21–69. Available: http://www.hscic.gov.uk/catalogue/PUB13218/HSE2012-Ch2-Phys-act-adults.pdf.

26. Cleland V, Dwyer T, Venn A. Which domains of childhood physical activity predict physical activity in adulthood? A 20-year prospective tracking study. Brit J Sport Med 2012;46:595-602.

27. Walker M, Shaper AG, Cook DG. Non-participation and mortality in a prospective study of cardiovascular disease. J Epidemiol Community Health 1987;41:295-9.

For peer review only

258

For peer review only

Supplementary table 1. Stability of physical activity variables over time by changes in working status (n=3288)

	Wave 1 to 2	Wave 1 to 3	Wave 1 to 4
	Kappa	Kappa	Kappa
**Total Physical Activity**			
No change in working status	0.28	0.18	0.24
Retired between follow ups	0.25	0.25	0.25
**Sport participation**			
No change in working status	0.36	0.32	0.33
Retired between follow ups	0.40	0.35	0.35
**Recreational activity**			
No change in working status	0.26	0.18	0.27
Retired between follow ups	0.22	0.19	0.14
**Walking**			
No change in working status	0.18	0.16	0.13
Retired between follow ups	0.12	0.09	0.11

Note. Kappa statistics are presented for participants with a valid physical activity score at all four time points and valid data on working status n=3288. 46.4% (n=1526) of men retired between wave 1 and 2; 71.5% (n=2352) of men retired between wave 1 and 3; and 79.4% (n=2611) were retired between wave 1 and 4.

For peer review only

STROBE Statement—Checklist of items that should be included in reports of ***cohort studies***

	Item No	Recommendation
**Title and abstract**	1	(*a*) Indicate the study's design with a commonly used term in the title or the abstract<br>Page 1 - 2
		(*b*) Provide in the abstract an informative and balanced summary of what was done and what was found<br>Page 2-3
**Introduction**		
Background/rationale	2	Explain the scientific background and rationale for the investigation being reported<br>Page 4-5
Objectives	3	State specific objectives, including any prespecified hypotheses<br>Page 5
**Methods**		
Study design	4	Present key elements of study design early in the paper<br>Page 5
Setting	5	Describe the setting, locations, and relevant dates, including periods of recruitment, exposure, follow-up, and data collection<br>Page 5
Participants	6	(*a*) Give the eligibility criteria, and the sources and methods of selection of participants. Describe methods of follow-up<br>Page 5
		(*b*) For matched studies, give matching criteria and number of exposed and unexposed<br>N/A
Variables	7	Clearly define all outcomes, exposures, predictors, potential confounders, and effect modifiers. Give diagnostic criteria, if applicable<br>Page 6-7
Data sources/ measurement	8*	For each variable of interest, give sources of data and details of methods of assessment (measurement). Describe comparability of assessment methods if there is more than one group<br>Page 6-7
Bias	9	Describe any efforts to address potential sources of bias<br>Page 7-8
Study size	10	Explain how the study size was arrived at<br>Page 8-9
Quantitative variables	11	Explain how quantitative variables were handled in the analyses. If applicable, describe which groupings were chosen and why<br>Page 6-7
Statistical methods	12	(*a*) Describe all statistical methods, including those used to control for confounding<br>Page 7-8
		(*b*) Describe any methods used to examine subgroups and interactions<br>Page 8
		(*c*) Explain how missing data were addressed<br>Page 8
		(*d*) If applicable, explain how loss to follow-up was addressed<br>Page 8 and 18
		(*e*) Describe any sensitivity analyses<br>Page 8

For peer review only - http://bmjopen.bmj.com/site/about/guidelines.xhtml

**Results**		
Participants	13*	(a) Report numbers of individuals at each stage of study—eg numbers potentially eligible, examined for eligibility, confirmed eligible, included in the study, completing follow-up, and analysed<br>Page 8 and 12
		(b) Give reasons for non-participation at each stage<br>Page 8
		(c) Consider use of a flow diagram<br>N/A
Descriptive data	14*	(a) Give characteristics of study participants (eg demographic, clinical, social) and information on exposures and potential confounders<br>Table 1
		(b) Indicate number of participants with missing data for each variable of interest<br>See table footnotes
		(c) Summarise follow-up time (eg, average and total amount)<br>Page 5
Outcome data	15*	Report numbers of outcome events or summary measures over time<br>Page 5
Main results	16	(*a*) Give unadjusted estimates and, if applicable, confounder-adjusted estimates and their precision (eg, 95% confidence interval). Make clear which confounders were adjusted for and why they were included<br>Tables 3 and 4
		(*b*) Report category boundaries when continuous variables were categorized<br>Page 6-7
		(*c*) If relevant, consider translating estimates of relative risk into absolute risk for a meaningful time period<br>N/A
Other analyses	17	Report other analyses done—eg analyses of subgroups and interactions, and sensitivity analyses<br>Page 11
**Discussion**		
Key results	18	Summarise key results with reference to study objectives<br>Page 15-16
Limitations	19	Discuss limitations of the study, taking into account sources of potential bias or imprecision. Discuss both direction and magnitude of any potential bias<br>Page 18
Interpretation	20	Give a cautious overall interpretation of results considering objectives, limitations, multiplicity of analyses, results from similar studies, and other relevant evidence<br>Page 17-19
Generalisability	21	Discuss the generalisability (external validity) of the study results<br>Page 18
**Other information**		
Funding	22	Give the source of funding and the role of the funders for the present study and, if applicable, for the original study on which the present article is based<br>Page 19

*Give information separately for exposed and unexposed groups.

For peer review only

Note: An Explanation and Elaboration article discusses each checklist item and gives methodological background and published examples of transparent reporting. The STROBE checklist is best used in conjunction with this article (freely available on the Web sites of PLoS Medicine at http://www.plosmedicine.org/, Annals of Internal Medicine at http://www.annals.org/, and Epidemiology at http://www.epidem.com/). Information on the STROBE Initiative is available at http://www.strobe-statement.org.

For peer review only

For peer review only - http://bmjopen.bmj.com/site/about/guidelines.xhtml
